# Enhancement of radiation therapy by indoleamine 2,3 dioxygenase 1 inhibition through multimodal mechanisms

**DOI:** 10.1186/s12885-023-10539-5

**Published:** 2023-01-18

**Authors:** Hiroaki Nozawa, Tetsuro Taira, Hirofumi Sonoda, Kazuhito Sasaki, Koji Murono, Shigenobu Emoto, Yuichiro Yokoyama, Yuzo Nagai, Shinya Abe, Soichiro Ishihara

**Affiliations:** Department of Surgical Oncology, The University of Tokyo, 7-3-1 Hongo, Bunkyo-Ku, Tokyo, 113-8655 Japan

**Keywords:** Indoleamine 2,3 dioxygenase 1, Colorectal cancer, Radiation therapy, Wnt/β-catenin pathway

## Abstract

**Background:**

Indoleamine 2,3-dioxygenase 1 (IDO1) is an enzyme that converts tryptophan to kynurenine. IDO1 expression is found not only in tumor cells but also in immune cells and is associated with tumor proliferation and immune responses. IDO1 inhibitors and radiation may cooperatively suppress tumor proliferation through the alterations in the Wnt/β-catenin pathway, cell cycle, and immune response. We investigated the antitumor effects of combination therapy of an IDO1 inhibitor, 1-methyl tryptophan (1-MT), and radiation on colorectal cancer.

**Methods:**

In vitro experiments were conducted using human and murine colon cancer cell lines (HCT116, HT-29, and Colon26). Cell growth inhibition was assessed using a MTS assay and Clonogenic assay. Cells were cultured for 48 h with or without 500 µM 1-MT after exposure to radiation (4 Gy). Cell cycle effects and modulation of Wnt/β-catenin pathway were evaluated using western blot analysis, flow cytometry, RT-PCR. Subcutaneous Colon26 tumors in BALB/c mice were treated by oral 1-MT (6 mg/mL) for 2 weeks and/or local radiation (10 Gy/10 fr). Bromodeoxyuridine (BrdU) incorporation in tumor cells and expression of differentiation markers of immune cells were evaluated using immunohistochemistry.

**Results:**

1-MT and a small interfering RNA against IDO1 suppressed proliferation of all cell lines, which was rescued by kynurenine. Clonogenic assay showed that administration of 1-MT improved radiosensitivity by suppressing the Wnt/β-catenin pathway activated by radiation and enhancing cell cycle arrest induced by radiation. Combination therapy showed a further reduction in tumor burden compared with monotherapies or untreated control, inducing the highest numbers of intratumoral CD3 + and CD8 + T cells and the lowest numbers of Foxp3 + and BrdU-positive tumor cells.

**Conclusions:**

The combination of 1-MT and radiation suppressed colon cancer cells in vitro and in vivo via multiple mechanisms.

**Supplementary Information:**

The online version contains supplementary material available at 10.1186/s12885-023-10539-5.

## Background

Preoperative chemoradiation therapy (CRT) is widely used as a standard therapy for patients with locally advanced lower rectal cancer [1]. Preoperative CRT can decrease the frequency of local recurrence and improve sphincter preservation rates with reduced side effects than postoperative CRT [2–5]. Prognosis of patients with a pathological complete response (pCR), defined as no remaining tumor at pathological examination, is known to be excellent [6]. However, only 10–20% of patients can achieve a pCR by conventional fluoropyrimidine‑based CRT [7, 8], and there is no evidence that CRT leads to improved overall survival [9]. Hence, novel therapeutic regimens for improving radiosensitivity of tumor are being investigated worldwide. Several trials on the effect of adding oxaliplatin to conventional fluoropyrimidines‑based CRT have been reported, although their conclusions regarding prognoses were inconsistent [10–14]. Our group reported that the pCR rate increased to 22.7% by adding irinotecan (80 mg/m^2^ every 2 weeks for 1 cycle) to conventional CRT [15]. In contrast, the ARISTOTLE trial reported that the addition of irinotecan (60 mg/m^2^ every week for 1 cycle) did not significantly improve the pCR rate [16]. Therefore, the benefit of adding irinotecan to CRT has not been fully proven.

Enhancing response to radiation therapy is multimodal and associated with numerous biological and genetic alterations [17]. First, radiation induces cell cycle arrest at the G2/M phase [17, 18]. Second, tumor cell death caused by radiation results in the release of cellular components which recruits dendric cells and activates effector T cells [19]. In contrast, repeated irradiation was shown to result in the enhancement of the Wnt/β-catenin pathway, increasing radioresistance in human colorectal cancer cell lines [20]. Therefore, we hypothesized that the inhibition of the Wnt/β-catenin pathway upregulated by radiation may improve the therapeutic efficacy of radiotherapy.

Indoleamine 2,3-dioxygenase 1 (IDO1) is an enzyme that converts tryptophan to kynurenine [21]. IDO1 is induced by inflammatory cytokines such as interferon γ (IFN γ) and TNF α [21]. In tumor cells, kynurenine accentuates the cell cycle, and IDO1 inhibition significantly reduces the number of cells in S phase [22]. IDO1 expression in dendritic cells was shown to decrease local tryptophan concentrations and inhibit T cell proliferation [23]. In effector T cells, tryptophan metabolism activates the general control non-depressive 2 kinase (GCN2) and causes T cell arrest at G1 phase [24]. On the other hand, kynurenine activates the aryl hydrocarbon receptor (AHR) and converts naïve CD4 + T cells to regulatory T cells [25]. These regulatory T cells in turn induce more IDO1-positive dendritic cells through the cytotoxic T lymphocyte-associated antigen-4 (CTLA-4) through a positive feedback mechanism [26]. Moreover, in an in vitro experiment, IDO1 was shown to upregulate cell proliferation by promoting the accumulation of β-catenin in the nucleus and activating the Wnt/β-catenin pathway [22]. In a clinical study, IDO1 was expressed in some patients with melanoma, lung, breast, esophagus, stomach, or colorectal cancers [27]. The expression of IDO1 in colorectal cancer as well as other malignancies is associated with poor prognosis [28, 29].

Based on the above-mentioned findings, we hypothesized that IDO1 inhibition and radiation could work cooperatively to induce cell cycle arrest and accentuate the antitumor immune response. Moreover, we anticipated that IDO1 inhibition may reverse upregulation of the Wnt/β-catenin pathway caused by radiation. In the current study, we conducted in vitro and in vivo experiments to investigate the antitumor effects of combining an IDO1 inhibitor with radiation for colorectal cancer.

## Methods

### Cell culture and reagents

Human colorectal cancer cell lines HCT116 and HT-29 and murine colorectal cancer cell line Colon26 were obtained from the Japanese Cancer Research Resources Bank. All cells were cultured in RPMI 1640 medium (Sigma Aldrich, St. Louis, MO) with 1% antibiotic/antimycotic solution (Thermo Fisher Scientific, Waltham, MA) and 10% fetal bovine serum (FBS; Atlas Biologicals, Fort Collins, CO) in a 5% CO_2_ incubator at 37℃. IDO1 inhibitor, 1-methyl tryptophan (1-MT; Sigma Aldrich), was dissolved in phosphate-buffered saline (PBS) for the in vitro experiments. For the in vivo experiments, 1-MT was dissolved in 10 mmol/L NaOH in drinking water at a concentration of 6 mg/mL, and supplemented with Stevia sweetener (0.1%) to improve acceptance by mice.

### Small interfering RNA (siRNA)

Stealth RNAi Pre-Designed siRNA targeting IDO1 was synthesized by Thermo Fisher Scientific. The siRNA sequence was 5’-UUUGCAUUGCCUUGAAUACAGUAGG -3’. HCT116 and HT-29 cells were transfected with 10 nmol/L siRNA or control siRNA (siRNA Negative Control Med GC Duplex #2, Thermo Fisher Scientific) in a 0.6% RNAiMAX reagent (Thermo Fisher Scientific).

### Cell proliferation assay

HCT116, HT-29, and Colon26 cells were seeded in 96-well plates at a density of 5 × 10^3^ cells per well and incubated for 48 h or 96 h with or without 1-MT (100, 500, or 1000 µM). All cells were also treated with 1-MT (500 µM) with or without the administration of kynurenine (50, 100, or 200 µM). In addition, HCT116 and HT-29 cells were incubated for 48 h with siRNA with or without the administration of kynurenine (100 µM). Then, cells were incubated with MTS reagent (Promega Corporation, Madison, WI) for 3 h, and absorbance at 490 nm was measured using a plate reader (InterMed, Tokyo, Japan). The values are expressed as means ± standard deviations (SD).

### Drug treatment and exposure to radiation

Cells were treated with 1-MT (500 µM) 1 h prior to radiation therapy (RT). Irradiation was performed sing an X-ray generator (Pantac HF350; Kyoto, Japan) at doses of 0, 2, 4, and 6 Gy (1.0 Gy/min).

To evaluate the effects of 1-MT and/or radiation on Wnt/β-catenin pathway and cell cycle, cells were cultured for 48 h with or without 500 µM 1-MT after exposure to radiation (4 Gy). To assess the effect of IFNs on IDO1 expression, cells were cultured for 48 h after 1–100 ng/ml of IFN α (R&D Systems, Inc. Minneapolis, MN), 1–100 ng/ml of IFN β (Thermo Fisher Scientific), or 0.1–10 ng/ml of IFN γ (Sigma Aldrich). Cells were cultured for 48 h after exposure to radiation (4 Gy) in order to assess the effect of radiation on IFN expression.

### Clonogenic assay

Two hundred–one thousand two hundred cells were seeded per well in a 6-well plate. Cells were treated with or without 1-MT (500 µM) 1 h prior to irradiation (0, 2, 4, or 6 Gy). After incubation for 14 days, cells were fixed with 70% ethanol and visualized with 0.5% crystal violet solution. The number of colonies was counted, and the following formula was used to calculate survival fraction (SF): SF = number of colonies with radiation/number of colonies without radiation. For each experiment, colonies were counted from three different wells. All values are expressed as means ± SD.

### Western blot analysis

Western blot analysis was performed according to the following protocol [30]. Whole-cell lysate was extracted in Bolt LDS sample buffer (Life Technologies, Carlsbad, CA) and cOmplete ULTRA Tablets, Mini, EASYpack Protease Inhibitor Cocktail (Roche, Basel, Switzerland). Proteins were quantified using the Qubit protein assay kit (Life Technologies). Equal amounts of proteins (40 μg) were separated by sodium dodecyl sulfate‑polyacrylamide gel electrophoresis with a 4–12% Bis‑Tris gel (Thermo Fisher Scientific) and transferred to a polyvinylidene difluoride membrane using the iBlot 2 system (Life Technologies). The membrane was trimmed for only loaded lanes. After blocking in 5% nonfat dry milk in Tris-buffered saline with 0.05% Tween 20 (TBST) for 1 h, the membrane was incubated with primary antibodies diluted in blocking buffer overnight at 4 °C. The membrane was then incubated with horseradish peroxidase-conjugated donkey anti-rabbit IgG secondary antibodies (Cytiva, Buckinghamshire, UK) for 1 h at room temperature. The immunoreactive bands were detected using ECL Prime Western Blotting Detection Reagent (Cytiva) and Luminograph I (Atto Corporation, Tokyo, Japan).

The primary antibodies used for western blot analysis were anti-IDO1 (mIDO-48, 1:1000 dilution; Santa Cruz Biotechnology), anti-cyclin B1 (1:1000 dilution; Cell Signaling Technology), and anti-β-actin (1:1000 dilution; Medical and Biological Laboratories, Beverly, MA).

### Quantitative reverse-transcription polymerase chain reaction (RT-PCR)

RT-PCR was performed according to the following protocol and data was analyzed using the 2^−ΔΔCT^ method [31]. Total RNA was isolated from cell using the ReliaPrep RNA Miniprep Systems (Promega Corporation), and spectrophotometry was used to measure mRNA concentrations. Synthesis of cDNA was performed using the SuperScript IV First-Strand Synthesis System (Thermo Fisher Scientific). The PCR products were amplified based on the SYBR Green method using a StepOne Real Time PCR System (48-well format; Applied Biosystems).

The sequences of the forward and reverse primers are listed in S.Table 1. The following reaction protocols were applied: initial denaturation at 95 °C for 10 min followed by 40 cycles of 95℃ for 15 s, 56–60℃ for 30 s, and 72℃ for 45 s. The cycle threshold (CT) required for the fluorescence was determined for each sample as the average value from three independent reactions. The result for each gene was reported as fold change after normalization against *GAPDH*, as quantified using the following formula.

$$\Delta CT (sample) = CT (target gene) - CT (GAPDH)$$

$$\Delta\Delta CT = \Delta CT (target sample) - \Delta CT (control sample)$$

$$Fold change = 2 - \Delta\Delta CT$$

### Immunofluorescence microscopy

After fixation with 4% paraformaldehyde for 10 min, cells were permeabilized with 0.05% Triton X-100 in PBS for 10 min at room temperature. Cells were blocked with 3% bovine serum albumin (BSA) in PBS for 1 h, and incubated overnight at 4 °C with primary antibodies diluted in blocking buffer. Cells were then incubated for 1 h at room temperature with Alexa Fluor 488 conjugated goat anti-rat IgG secondary antibodies. Nuclear staining was performed using 4’,6-diamidino-2-phenylindole (DAPI) with VECTASHIELD Mounting Medium (Vector Laboratories, Burlingame, CA). Stained cells were observed under a fluorescence microscope (BZ-8100; Keyence, Osaka, Japan). The primary antibody used for immunofluorescence was anti-IDO1 (mIDO-48, 1:500 dilution; Santa Cruz Biotechnology).

### Cell cycle analysis

After labeling with 1 mM bromodeoxyuridine (BrdU) solution for 1 h, cells were fixed with BD Cytofix/Cytoperm Buffer (BD Pharmingen, San Diego, CA) and permeabilized with BD Cytoperm Plus Buffer (BD Pharmingen) on ice. Subsequently, cells were incubated with DNase for 1 h. Finally, BrdU and total DNA were stained with a 7-aminoactinomycin D (7-AAD) solution and a fluorescein isothiocyanate (FITC) anti-BrdU antibody in a shaded area. The percentage of cells in G1, S, and G2/M phase of the cell cycle was measured by flow cytometry. All values are expressed as means ± SD.

### Animal experiments

A total of 60 female BALB/c mice (4 weeks old) were purchased from Oriental Yeast Co., Ltd. (Tokyo, Japan) and bred under a constant temperature (20–24 °C) and humidity (45–65%) and a 12-h light–dark cycle with free access to food and water. Colon26 cells (5 × 10^5^ cells) were dissolved in serum-free RPMI 1640 medium and injected subcutaneously in the right flank of the mice. After two weeks, when their tumors grew to 100–200 mm^3^ in size, mice were divided into four treatment groups (each group: 15 mice) as follows: vehicle control, 1-MT alone (6 mg/mL in drinking water), radiation alone (10 Gy/10 fr), and combined therapy (1-MT + RT). Mice in cages received the same treatments. Radiation was delivered five times weekly with a daily fraction of 1 Gy for 2 weeks (on days 1 to 5 and days 8 to 12) after shielding the whole body with lead except for the tumor. Data of all mice were not blinded and subjected to the following analyses.

The longest and shortest diameters of the implanted tumors were measured three days a week, and the tumor volumes were calculated using the following formula: 0.5 × (shortest diameter)^2^ × (longest diameter) [32]. The body weights of the mice were also measured.

After treatment, mice were euthanized by intraperitoneal injection of thiopental sodium (50 mg/kg) and rapid cervical dislocation. Death was verified by monitoring breathing, heartbeat, flexor reflex and corneal contact response. The subcutaneous tumors, liver, lung, heart, kidney, and spleen were resected and stored as paraffin-embedded sections for immunohistochemistry.

### Immunohistochemistry of in vivo tumors

Paraffin-embedded formalin-fixed Sects. 5 μm in thickness were deparaffinized in xylene and then rehydrated in ethanol. The sections were immersed in citrate buffer and autoclaved at 121 °C for 10 min for antigen retrieval. Endogenous peroxidase activity was blocked with 0.3% hydrogen peroxide in methanol for 30 min, and the sections were incubated with 1% BSA in PBS for 1 h at room temperature. After overnight incubation with the primary antibody at 4 °C, the sections were anti-rabbit IgG antibody (Nichirei Biosciences Inc., Tokyo, Japan) at room temperature for 20 min. The enzymatic reaction of horseradish peroxidase labeled with secondary antibody was detected by the substrate 3,3’-diaminobenzidine (DAB; FUJIFILM Wako Pure Chemical Corp., Osaka, Japan). Counterstain was performed using hematoxylin. The primary antibodies used were anti-CD8 (1:100 dilution; Cell Signaling Technology), anti-CD3 (1:100 dilution; Cell Signaling Technology), anti-Foxp3 (1:100 dilution; Cell Signaling Technology), anti-CD68 (1:100 dilution; Cell Signaling Technology), anti-Ly6G (1:100 dilution; Cell Signaling Technology), and anti-CD31 (1:100 dilution; Santa Cruz Biotechnology).

The numbers of stained immune and vascular endothelial cells were counted in two fields from five tumors for each treatment group under 400 × magnification, and the number of vascular endothelial cells in a field was defined as the microvessel density (MVD).

### Bromodeoxyuridine incorporation assay of in vivo tumors

BrdU (50 mg/g of body weight) were injected intraperitoneally to mice 1 h before sacrifice. Dissected tumors were subjected to immunohistochemical staining. The primary antibody used was a rat anti-BrdU antibody (Abcam, Cambridge, UK) and the reaction was visualized using DAB. The number of cells positive for BrdU was counted on images captured under 400 × magnification in three fields from five tumors for each treatment group.

### Biochemical analysis

After the final day of treatment, blood was collected from three mice in each treatment group. After euthanasia, blood was obtained by puncture axillary artery and vein. The blood volume collected from each mouse was 500 μl. Death was verified as described above. The numbers of white blood cells (WBC), red blood cells (RBC), and platelet (Plt) and hemoglobin (Hb) were measured using an automatic blood cell counter (Celltac alpha; Nihon Kohden, Tokyo, Japan). Serum levels of aspartate aminotransferase (AST), alanine aminotransferase (ALT), alkaline phosphatase (ALP), and creatinine (Cre) were measured using BiOLiS 24i (Tokyo Boeki Medisys Inc., Tokyo, Japan). Coagulated samples were excluded from the a Line 7nalysis.

### Statistical analysis

All experiments were performed repeatedly at least three times. The statistical significance of differences was evaluated by the unpaired, two-tailed Student’s *t*-test. All analyses were performed using the JMP Pro 16.2 software (SAS Institute Inc, Cary, NC), with *p* < 0.05 considered significant. Bonferroni correction was used during multiple comparison testing, and *p* < 0.0083 was considered significant.

## Results

### IDO1 inhibition suppressed proliferation of the colorectal cancer cell lines

**Fig. 1:**
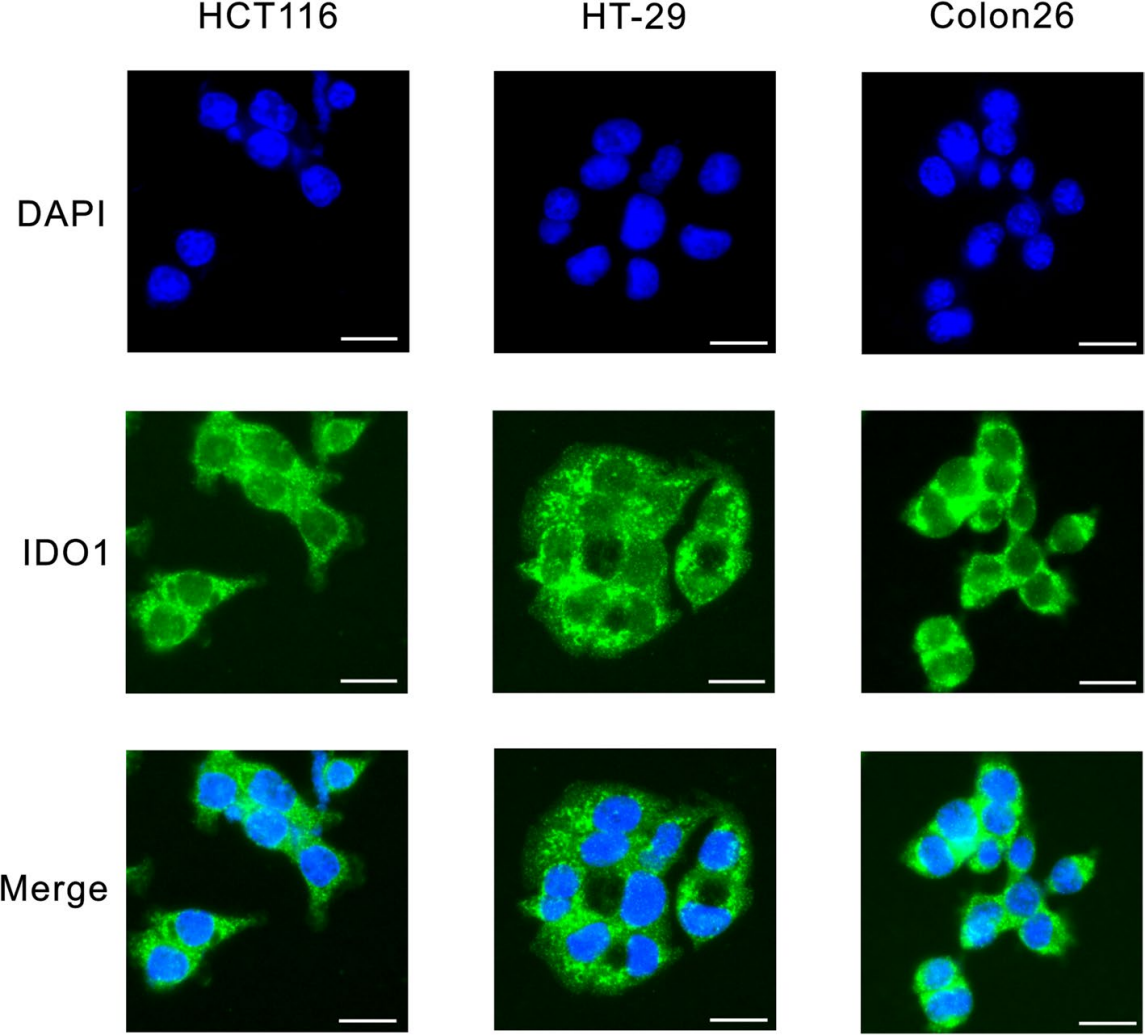
IDO1 expression in colorectal cancer cell lines. IDO1 was stained by green fluorescence, and the nuclei were visualized blue with DAPI. Bars, 20 µm

IDO1 was expressed in the cytoplasm of HCT116, HT-29, and Colon26 cells as revealed by immunofluorescence (Fig. 1).

**Fig. 2:**
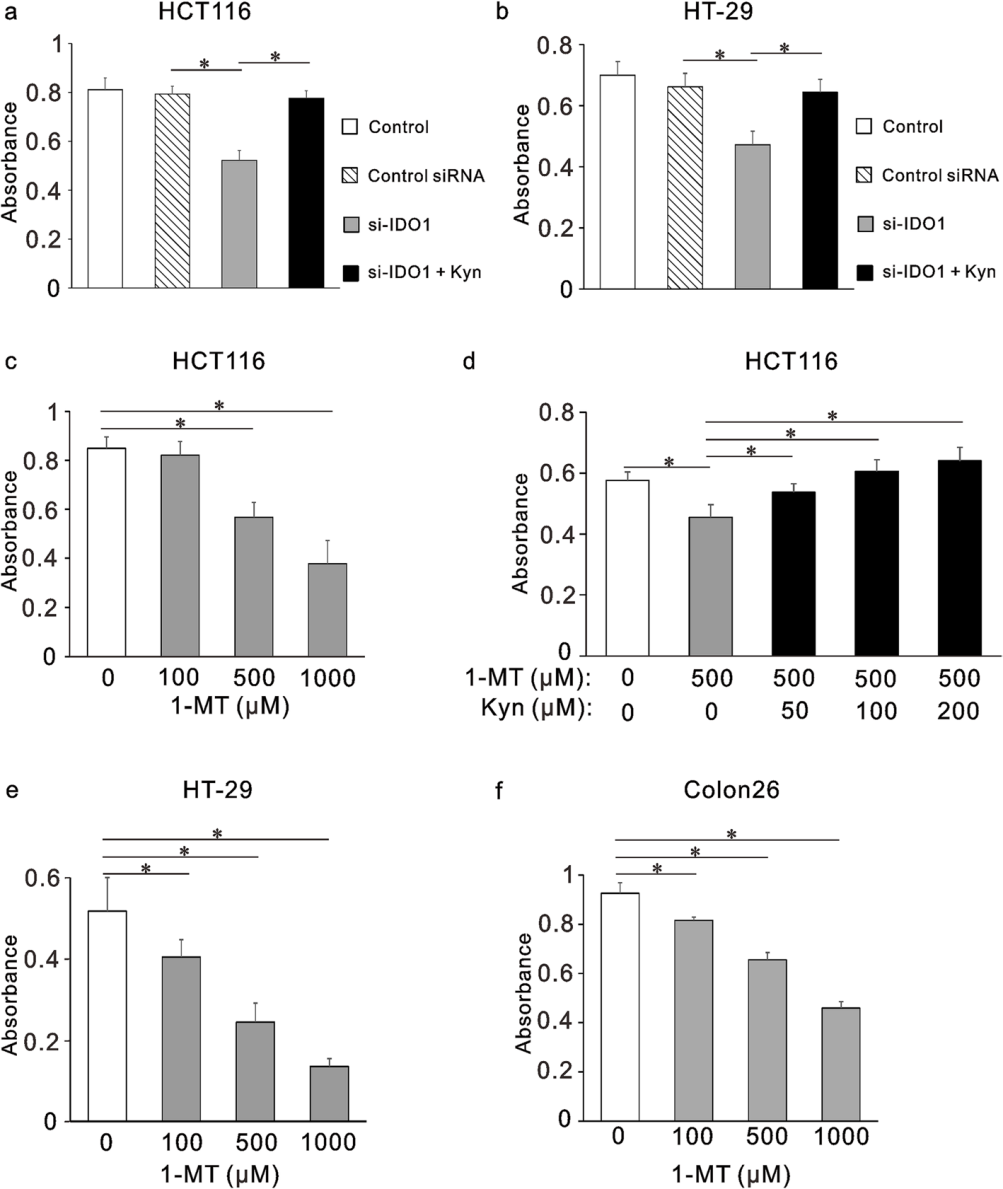
Effects of IDO1 inhibition on the proliferation of colorectal cancer cells in vitro*.* Inhibition by gene silencing of *IDO1* in HCT116 (**a**) and HT-29 cells (**b**). Inhibition by 1-methyl tryptophan (1-MT) in HCT116 cells (**c,d**), HT-29 (**e**) and Colon26 (**f**). Results after 48-h treatment are shown as absorbances measured at 490 nm by the MTS assay. Concentration of siRNA,10 nM; concentration of kynurenine (Kyn), 100 µM. Bars indicate standard deviations. * *p* < 0.001

The effect of IDO1 inhibition on the proliferation of colorectal cancer cells was investigated using MTS assay. siRNAs against the *IDO1* gene decreased mRNA expression by 90% compared with untreated cells or control siRNA in HCT116 and HT-29 cells (*p* < 0.001, S.Fig. 1). The expression of IDO1 protein was also decreased by siRNAs against the *IDO1* gene (*p* < 0.001, S.Fig. 1). As shown in Fig. 2a and b, gene silencing of *IDO1* significantly inhibited cell growth in HCT116 and HT-29 cells (*p* < 0.001), while kynurenine completely rescued the proliferation of these knocked-down cells to the untreated levels. Similarly, 1-MT treatment for 48 h and 96 h decreased proliferation of HCT116, HT-29, and Colon26 cells in a dose-dependent manner (*p* < 0.001, Fig. 2c, e, f, and S.Fig. 2), and kynurenine rescued the proliferation of HCT116 cells treated with 1-MT to the untreated levels (Fig. 2d).

### 1-MT increased radiosensitivity in the colorectal cancer cell lines

**Fig. 3:**
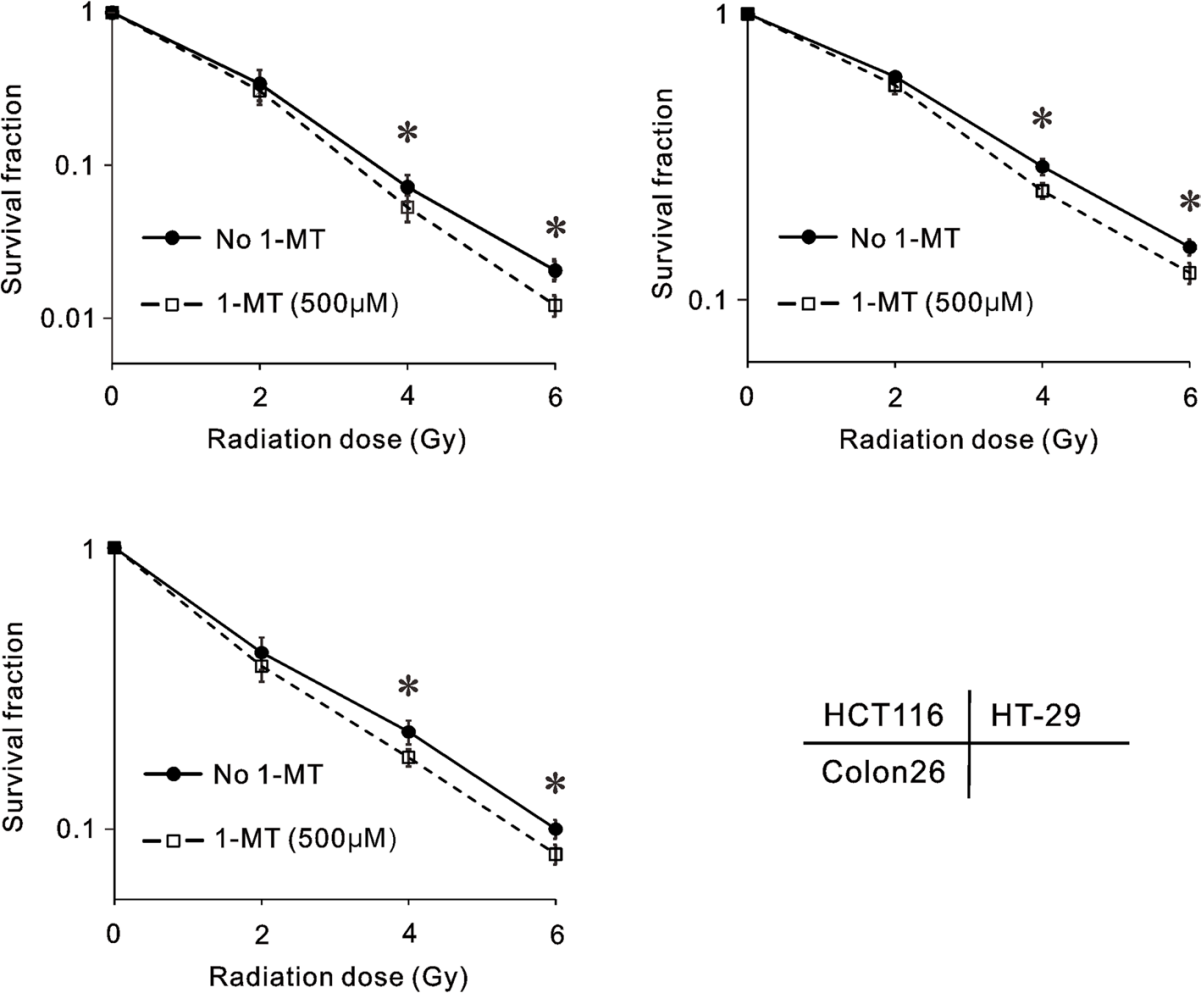
Clonogenic assay of colorectal cancer cells after exposure to various doses of radiation with or without 1-MT. HCT116 (upper left panel), HT-29 (upper right panel), and Colon26 (lower left panel). Bars indicate standard deviations. * *p* < 0.05 in comparison between cells treated with and without 1-MT (500 µM)

To evaluate whether IDO1 inhibition increases radiosensitivity in colorectal cancer cells, surviving cells treated by radiation with or without 1-MT were counted by clonogenic assay. Radiation alone reduced the SF of HCT116, HT-29, and Colon26 cells in a dose-dependent manner (*p* < 0.05), while 1-MT significantly enhanced the radiosensitivity of all cells at 4 and 6 Gy (Fig. 3).

### Wnt/β-catenin pathway modulation by 1-MT and radiation

**Fig. 4:**
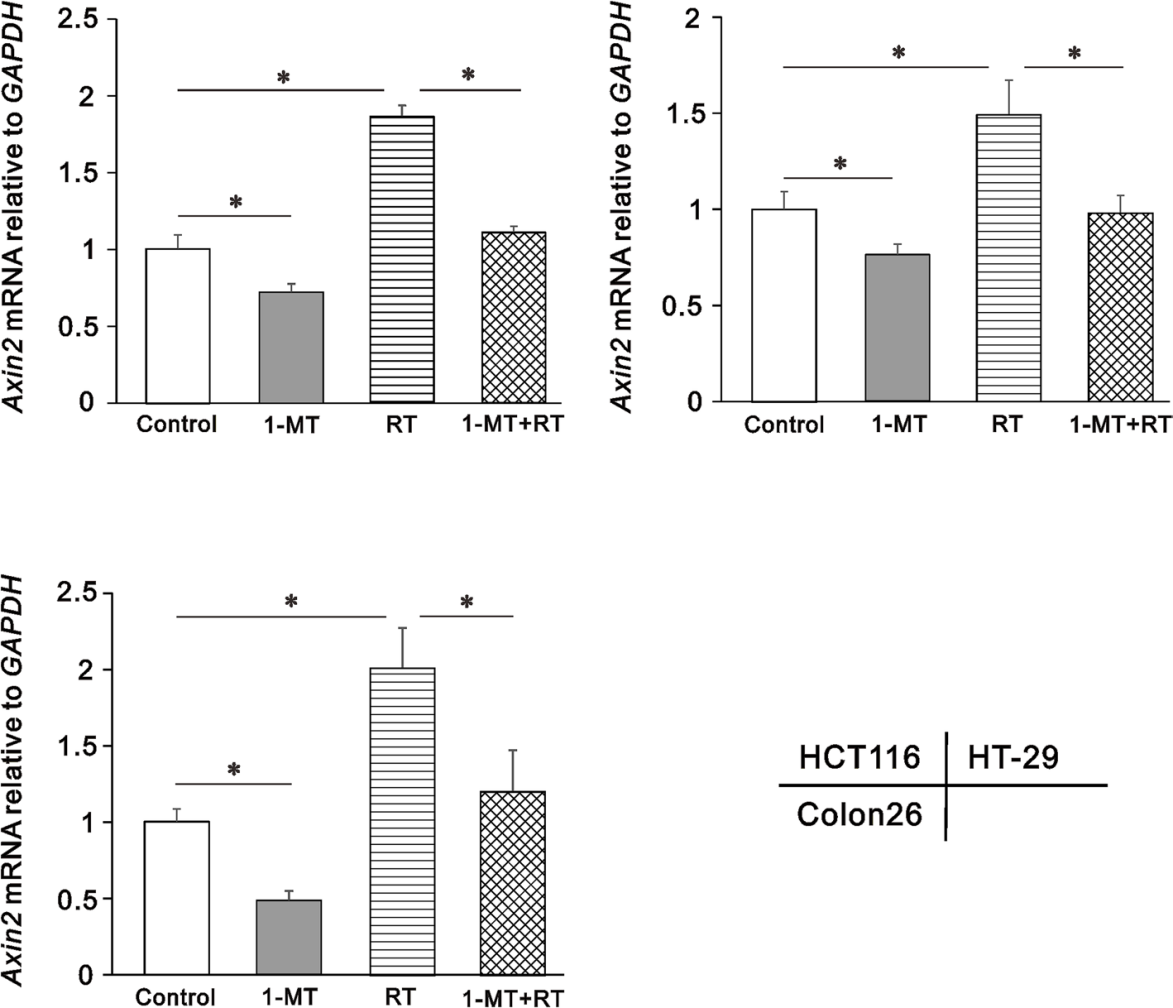
*Axin2* mRNA expression in colorectal cancer cells treated by 1-MT and/or radiation by quantitative reverse-transcription polymerase chain reaction. HCT116 (upper left panel), HT-29 (upper right panel), and Colon26 (lower left panel). RT: radiation. Results 48 h after administration of 1-MT (500 µM) and/or radiation (4 Gy) are shown. Bars indicate standard deviations. * *p* < 0.0083

To elucidate the changes in the Wnt/β-catenin pathway induced by 1-MT and radiation, we measured mRNA expression of *Axin2*, a downstream target of the pathway. In response to radiation, mRNA expression increased by 50–100% in all three cell lines, indicating that radiation activated the Wnt/β-catenin pathway (*p* < 0.0083, Fig. 4). In contrast, the expression levels of *Axin2* decreased after treatment with 1-MT, and, moreover, the elevated *Axin2* mRNA expression following radiation treatment was reduced to the untreated levels after administration of 1-MT.

### Inhibition of the colorectal cancer cell cycle by 1-MT and radiation

**Fig. 5:**
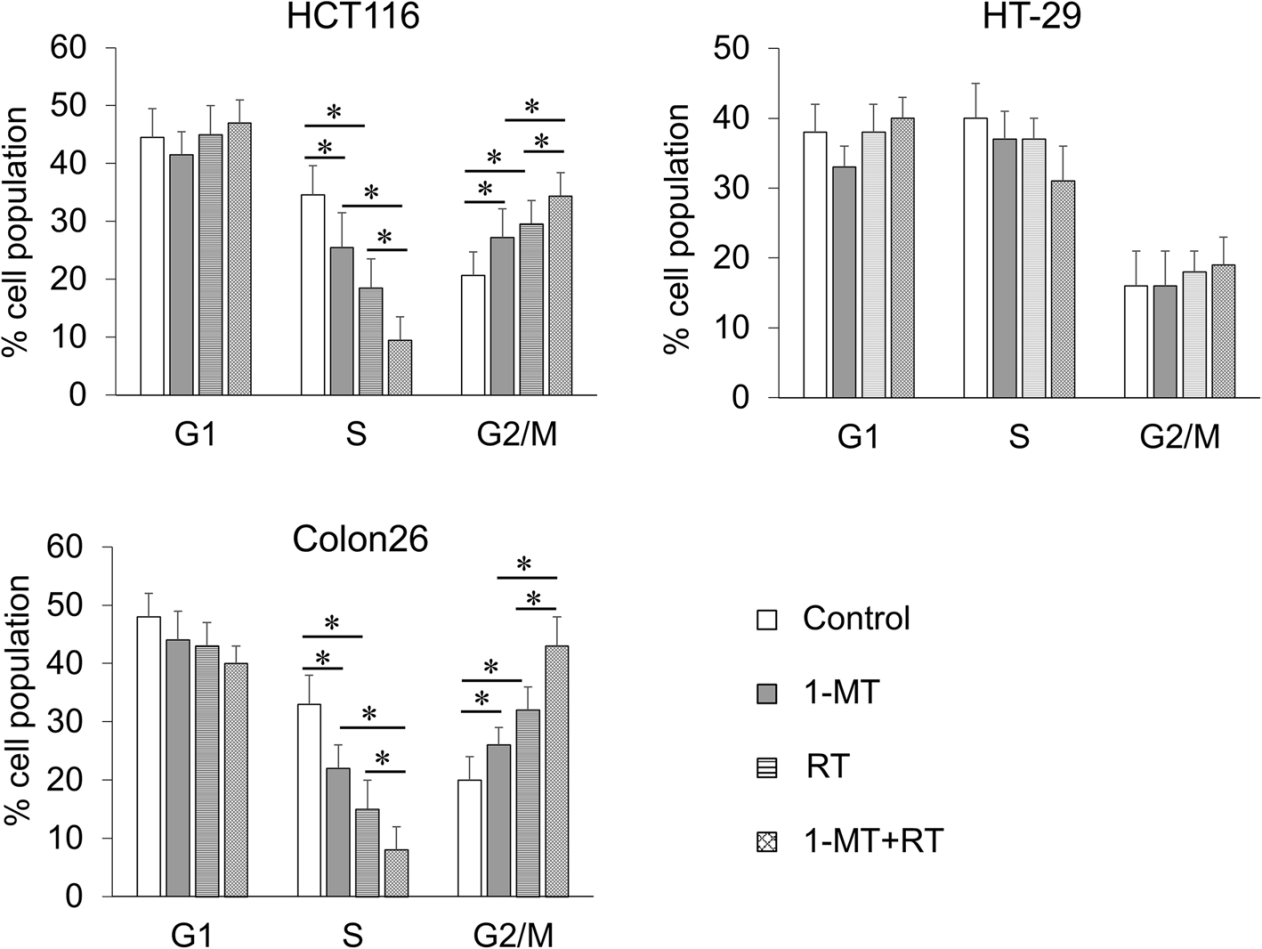
Cell cycle analysis of colorectal cancer cells treated by 1-MT and/or radiation by flow cytometry. HCT116 (upper left panel), HT-29 (upper right panel), and Colon26 (lower left panel). RT: radiation. Results 48 h after administration of 1-MT (500 µM) and/or radiation (4 Gy) are shown. Bars indicate standard deviations. * *p* < 0.0083

To determine whether the inhibitory effect of 1-MT and radiation on cell proliferation is related to the cell cycle arrest, we performed cell cycle analysis on 1-MT-treated and irradiated cells. As shown in Fig. 5, 1-MT treatment of HCT116 and Colon26 cells resulted in cell cycle arrest at the G2/M phase (HCT116; *p* = 0.007, Colon26; *p* = 0.005), radiation treatment of HCT116 and Colon26 cells also resulted in cell cycle arrest at the G2/M phase (HCT116; *p* < 0.001, Colon26; *p* < 0.001), whereas cell cycle arrest was not observed in HT-29 cells. Furthermore, combination therapy enhanced cell cycle arrest at the G2/M phase and decreased the population of cells in S phase (*p* < 0.001). The fraction of cells in G1 phase was essentially unchanged across the cell lines.

To examine the molecular mechanism of cell cycle arrest at the G2/M phase by 1-MT and radiation, we analyzed cyclin B1 protein expression. HCT116 and Colon26 cells treated with a combination of 1-MT and radiation showed a marked reduction in cyclin B1 expression compared to untreated cells or those treated with 1-MT or radiation alone (S.Fig. 3). In contrast, these changes were not observed in HT29 cells.

### Interaction between radiation exposure and IFN that induce IDO1

As shown in S.Fig. 4, IFN γ increased the mRNA expression of *IDO1* in a dose-dependent manner in HCT116, HT-29, and Colon26 cells (*p* < 0.001). In contrast, IFN α and IFN β had no effect on the mRNA expression of *IDO1*.

In response to radiation, both *IFN α* and *IFN β* mRNA increased in HCT116, HT-29, and Colon26 cells (*p* < 0.001, S.Fig. 5). Meanwhile, *IFN γ* mRNA was not detected irrespective of exposure to radiation in these cell lines.

### 1-MT and radiation reduced tumor burden in mouse allografts

**Fig. 6:**
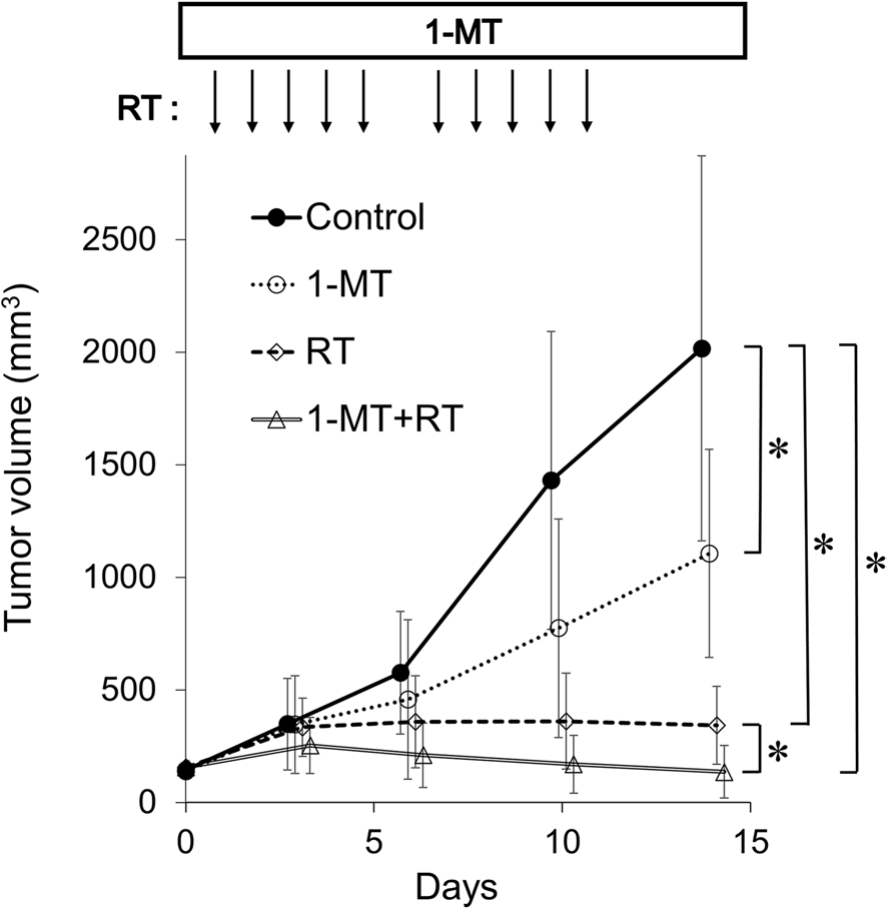
Effects of 1-MT and/or radiation on subcutaneous Colon26 tumors. Temporal change in tumor size over treatment course was shown for each group. RT: radiation. Bars indicate standard deviations. * *p* < 0.001

Next, the antitumor effects of 1-MT and radiation were evaluated using mouse subcutaneous colorectal tumor models. Oral 1-MT tended to suppress the volume of Colon26-derived tumors after treatment for 2 weeks (*p* = 0.02). Local radiation (10 Gy/10 fr) significantly suppressed tumor growth (*p* < 0.001). The combination of 1-MT and radiation markedly reduced tumor volumes compared to the vehicle control or monotherapies (*p* < 0.001, Fig. 6). The mean tumor volume before treatment was 161 mm^3^. The mean tumor volumes on day 14 for the vehicle control, 1-MT alone, radiation alone, and combined therapy (1-MT + RT) were 2016 mm^3^, 1105 mm^3^, 343 mm^3^, and 136 mm^3^, respectively.

### Histological evaluation of subcutaneous tumors

**Fig. 7:**
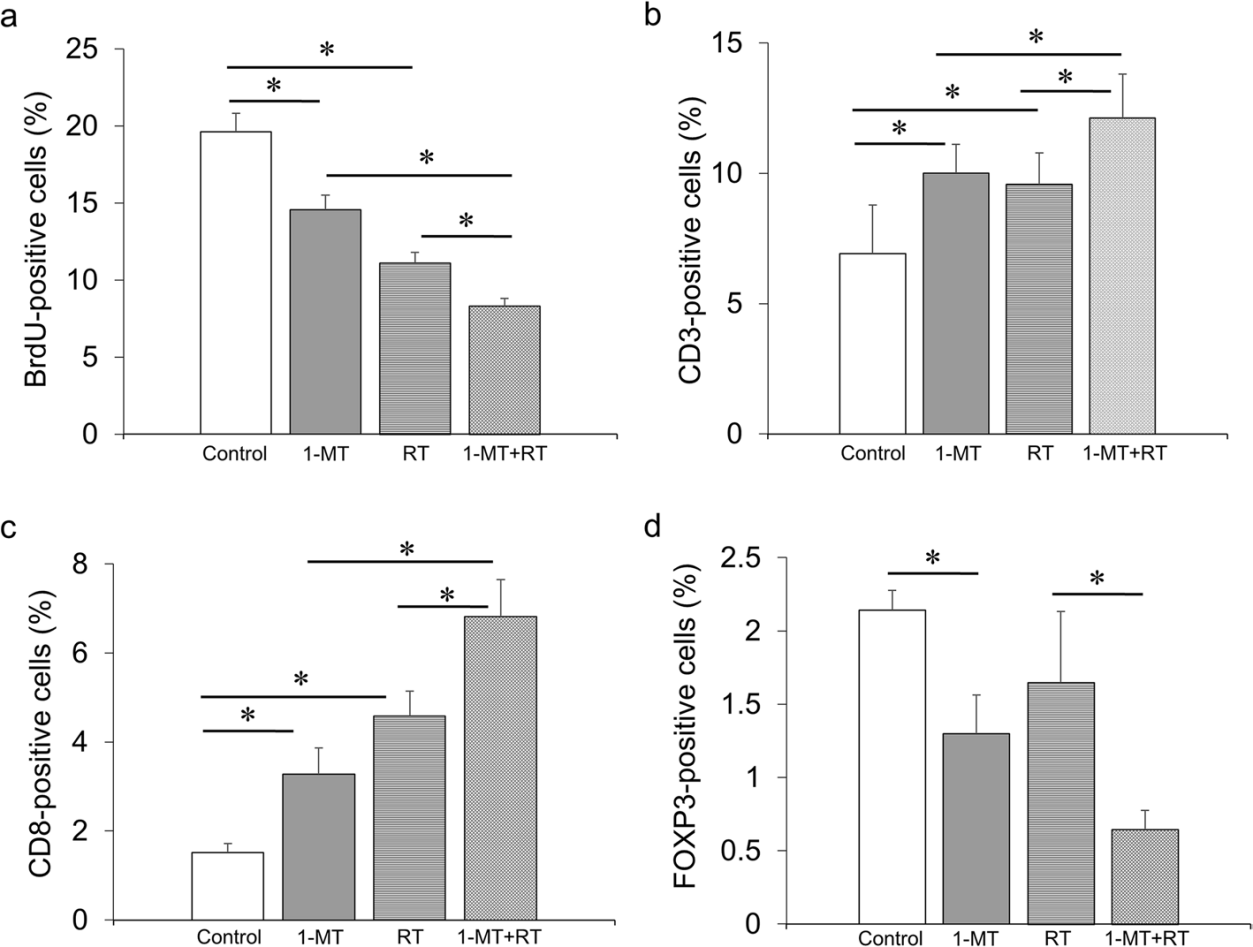
BrdU incorporation in tumor cells and expression of differentiation markers of immune cells in subcutaneous Colon26 tumors. **a** BrdU-positive tumor cells, **b** CD3 + T cells, **c** CD8 + T cells, **d** Foxp3 + T cells. RT: radiation. Treatments are shown as horizontal axis labels of the graph. Bars in graphs indicate standard deviations. * *p* < 0.001

BrdU incorporation of subcutaneous tumor specimens was evaluated. As shown in Fig. 7a, the number of BrdU-positive tumor cells in mice treated with 1-MT was significantly lower than the control (14.5% vs. 19.6%, *p* < 0.001), as was the case with radiation (11.1% vs. 19.6%, *p* < 0.001). Combination therapy further reduced the number of BrdU-positive cells (8.3%, *p* < 0.001).

Next, we examined tumor-associated lymphocytes and myeloid cells. 1-MT or radiation alone resulted in an increase in CD3 + T cells compared with the control (9.6% vs. 6.9%, *p* < 0.001; 10.0% vs. 6.9%, *p* < 0.001, respectively). The combination of 1-MT and radiation further increased the number of CD3 + T cells (12.1%, *p* < 0.001, Fig. 7b). Similarly, 1-MT or radiation increased the number of CD8 + T cells compared with the control (4.6% vs. 1.5%, *p* < 0.001; 3.3% vs. 1.5%, *p* < 0.001, respectively), and combination therapy further increased the number of CD8 + T cells (6.8%, *p* < 0.001, Fig. 7c). In contrast, radiation was not associated with the number of intratumoral Foxp3 + regulatory T cells (1.6% vs. 1.3%, *p* = 0.11), while Foxp3 + cells were markedly reduced in mice treated with 1-MT compared with the control (2.1% vs. 1.3%, *p* < 0.001). The reduction in Foxp3 + cells by 1-MT was also observed in irradiated mice (0.6% vs. 1.6%, *p* < 0.001; Fig. 7d). The number of CD68 + macrophages and Ly6G + granulocytes were similar among the four treatment groups (S.Fig. 6). Evaluation of tumor-associated microvessels by CD31 staining showed that the MVD was not affected by 1-MT or radiation (S.Fig. 7).

### Safety of combination therapy

**Fig. 8:**
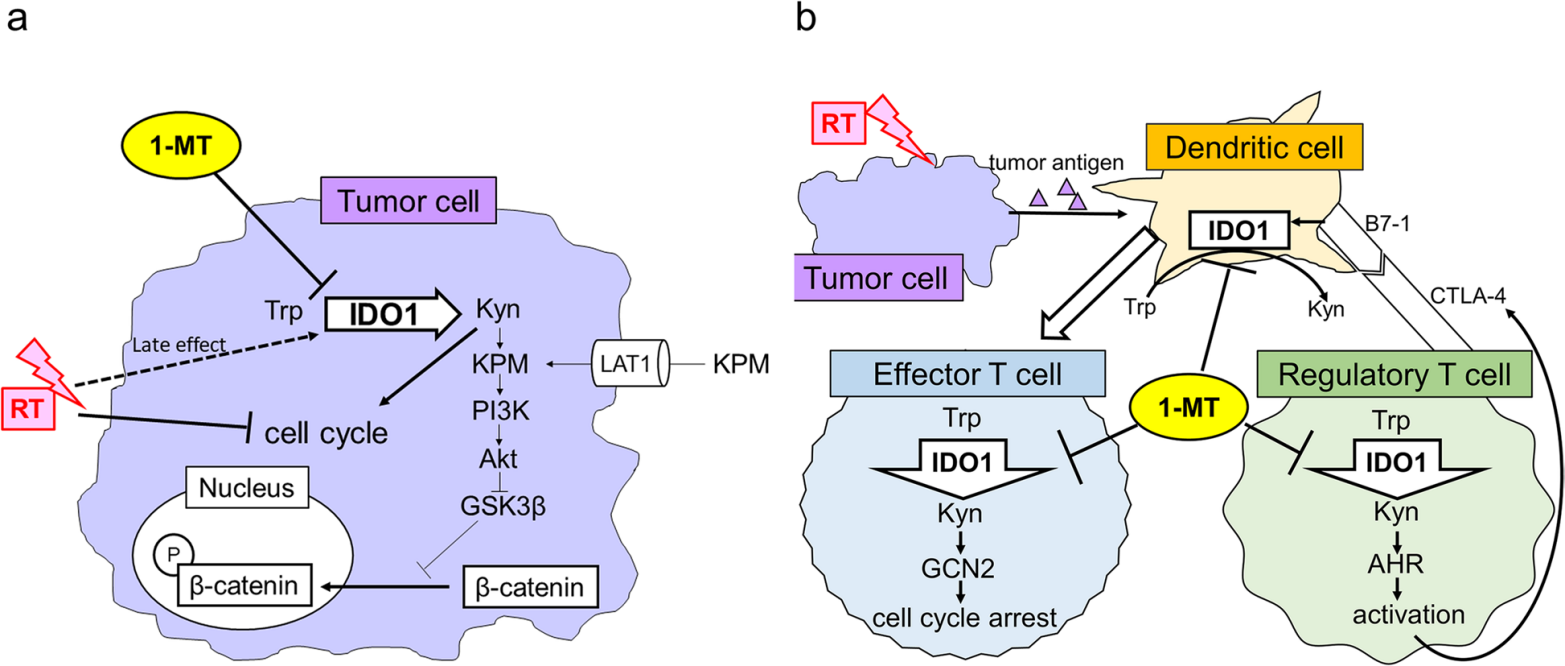
Schematic diagrams illustrating the mechanisms for tumor-suppression by IDO1 blockade and radiation. **a** Schematic model for IDO1-induced sequential activations of kynurenine-PI3K-Akt axis and Wnt/β-catenin pathway in tumor cells, **b** Presumable mechanisms for activation of antitumor immune response by IDO1 blockade and radiation therapy. 1-MT: 1-methyl tryptophan, RT: radiation, Trp: tryptophan, IDO1: indoleamine 2,3 dioxygenase 1, Kyn: kynurenine, KPM: kynurenine pathway metabolites, LAT1: L-amino acid transporter 1, CTLA-4: cytotoxic T lymphocyte-associated antigen-4, GCN2: general control non-depressive 2 kinase, AHR: aryl hydrocarbon receptor

The body weights of mice were similar among the treatment groups (S.Fig. 8). Blood tests indicated that while radiation induced leukocytopenia, 1-MT did not affect the WBC count. Anemia was not observed in any of the treatment groups, and there were no obvious differences in platelet counts or serum levels of AST, ALT, ALP, and Cre among the treatment groups (S.Fig. 9). We also did not detect obvious histological changes in the liver, lung, heart, kidney, or spleen following the 1-MT and/or radiation treatments (S.Fig. 10).

## Discussion

Several IDO1 inhibitors have been developed for clinical use, including 1-MT, epacadostat, and navoximod. Phase I/II trials using these IDO1 inhibitors were conducted, but no trials using monotherapy treatments reported objective responses [33]. Therefore, recent clinical trials of IDO1 inhibitors have used them as combination therapies with other immunotherapy or chemotherapy drugs, whereas only a few trials have investigated their effectiveness when combined with radiation [34, 35]. For example, a trial investigating malignant brain tumors in childhood reported that only six of 29 patients completed combination therapy of 1-MT, temozolomide, and radiation, with only three patients exhibiting symptomatic improvement by the triplet therapy [35]. However, there have been no clinical trials investigating the combination of an IDO1 inhibitor and radiation in colorectal cancer. In a recent preclinical study, epacadostat enhanced the antitumor effects of radiation on colorectal cancer, but the underlying molecular mechanisms were not addressed [36]. Here, we demonstrated the antitumor effects of 1-MT and radiation in both in vitro and in vivo experiments and investigated the associated molecular mechanisms.

Aberrant Wnt/β-catenin pathway plays a critical role in colorectal carcinogenesis [37]. Dong et al. reported that radiation induced nuclear accumulation of β-catenin and promoted invasive activity in a glioblastoma cell line [38]. In human colorectal cancer cell lines, cells which survive irradiation were shown to have activated the Wnt/β-catenin pathway and acquired radiation resistance [20]. Furthermore, a clinical study demonstrated that high expression of β-catenin in rectal cancer treated by CRT was associated with poor disease-free survival [39]. In line with these studies, we demonstrated that radiation upregulated gene expression of *Axin2* in HCT116, HT-29 and Colon26. Interestingly, 1-MT suppressed this activated Wnt/β-catenin pathway to untreated levels, which indicates that targeting of Wnt/β-catenin pathway with 1-MT may be ideal for combination with radiotherapy.

In the course of anti-tumor immune response, both tumor-promoting and anti-tumor cytokines are released in the tumor microenvironment [40]. Among them, IFN γ is an anti-inflammatory cytokine produced by NK cells, T helper 1 cells and CD8 + cytotoxic T lymphocytes, and can inhibit directly tumor cell proliferation, increase tumor antigenicity, and suppress angiogenesis [40]. Meanwhile, IFNγ acts as an upstream regulator of IDO1 which metabolizes tryptophan to kynurenine. Kynurenine is further metabolized to quilolinic acid through multiple pathways, and quilolinic acid is finally converted to nicotinamide adenine dinucleotide [41]. These kynurenine pathway metabolites (KPM) are also incorporated into tumor cells through L-amino acid transporter 1 (LAT1) which is frequently upregulated in various human cancers [42]. KPM activate PI3K, which phosphorylates Akt at Ser473 and Thr308 [43]. Phosphorylated Akt then inactivates GSK3β by inducing its phosphorylation at Ser 9 [44], which results in increased phosphorylation of β-catenin at Ser 552 and promotes β-catenin nuclear translocation [45]. Therefore, it is suggested that IFN γ and IDO1 may upregulate Wnt/β-catenin pathway presumably via the sequential activation of kynurenine-PI3K-Akt axis (Fig. 8a), although it was not examined in the current study. We found that exposure to radiation (4 Gy) did not induce IFN γ whereas only IFN γ, not IFN α or IFN β, induced IDO1 in HCT116, HT-20, and Colon26. These results indicated that the activation of Wnt/β-catenin pathway by radiation, as reflected by Axin-2 expression, was at least independent of IFN γ and IDO1 in these colorectal cancer cell lines.

We found that 1-MT and radiation cooperatively induced cell cycle arrest at the G2/M phase in HCT116 and Colon26 cells but not in HT-29 cells. Radiation-induced cell cycle arrest is mediated by the activation of p53 protein [18]. HCT116 and Colon26 cells represent wild-type *p53*, while HT-29 cells carry a *p53* mutation [46, 47]. Thus, the distinct status of the *p53* gene in these cells may explain the above results regarding cell cycle arrest by radiation among the cell lines. In accordance with our results showing that 1-MT induced G2/M arrest, the administration of anti-*IDO1* shRNA increased tumor cells in the G2/M phase in a human lung adenocarcinoma cell line [48]. Our observations suggest that 1-MT-induced G2/M arrest may be p53-dependent. Regarding the link between IDO1 and p53, Tang et al. demonstrated that IDO1 promoted cell migration and invasion, which was attenuated by wild-type p53 in lung cancer cell lines [49]. Further studies will be required to clarify the association between IDO1 and p53 in cell cycle regulation.

In our in vivo experiments, the combination of 1-MT and radiation showed the lowest tumor burden among the four treatment groups. Correspondingly, combination therapy resulted in the most pronounced decrease in BrdU-positive cells compared with monotherapies or the untreated control. The antiproliferative effects of 1-MT and radiation appeared to substantiate our in vitro findings. Furthermore, 1-MT and radiation reduced tumor size from the baseline level after a 2-week treatment period. It will be interesting to investigate whether the tumor size reduction lasts beyond the duration of the treatment period or whether longer treatment with 1-MT and radiation is required for sustained therapeutic efficacy.

Our group previously reported that radiation plus interleukin-2 suppressed not only local tumor proliferation but also reduced distant metastasis using a mouse subcutaneous colorectal cancer tumor model [50]. This abscopal effect was accompanied by an increased number of CD4 + splenocytes induced by combination therapy [50]. In the current study, both 1-MT and radiation cooperatively increased CD8 + effector T cells and inhibited Foxp3 + regulatory T cells in allografted tumors. A schema of possible mechanism of anti-tumor immune activities by 1-MT and radiation is shown based on previous reports and the present in vitro and in vivo experiments (Fig. 8b). Whether these alterations in tumor-associated lymphocyte subpopulations caused by combination therapy are accompanied by changes in the systemic immune response remains to be elucidated.

As IDO1 is one of enzymes that catalyze the first and rate-limiting step in the tryptophan-kynurenine signaling cascade, it is relevant to target IDO1 in cancer therapy. In addition, tryptophan breakdown triggered by IDO1 is reported to contribute to cancer-related anemia, fatigue, depression, and decreased quality of life [51–53]. On the other hand, tryptophan, kynurenine, and KPMs play a crucial role in health maintenance, and their abnormalities have been implicated in many other diseases such as inflammatory bowel disease, cardiovascular disease, kidney disease, osteoporosis, diabetes, and neurologic and psychiatric disorders including Parkinson disease, Hunchinton’s disease, Alzheimer’s disease and depression [53–55]. Therefore, there is a concern that IDO1 blockade in various types of treatment settings may disturb body homeostasis. In our preclinical model, the combination of 1-MT and radiation was well-tolerated, with radiation-induced leukocytopenia being the only observable adverse effect; 1-MT-dependent adverse effects were not observed. These findings are consistent with a previous clinical trial of 1-MT monotherapy for metastatic solid malignancy, which reported that only one out of 48 patients had a grade 3 adverse event [56]. It is of paramount importance to ensure the safety of patients receiving new treatments [57]. In this regard, 1-MT and radiation is considered a promising treatment combination, because 1-MT is already a clinically available drug with few side effects.

There are several limitations of the current study. First, the association between kynurenine-PI3K/Akt and Wnt/β-catenin pathways remains to be clarified by further in vitro studies. The primary target molecules of radiation which are responsible for these pathways and Axin-2 overexpression are not identified. We performed in vivo experiments using a mouse subcutaneous tumor model that differs from an autologous tumor model or clinical carcinogenesis in terms of tumor genetic and epigenetic heterogeneity, an organ-specific tumor microenvironment, and inherent biological differences between species. A six-to-eight-week interval between preoperative CRT and surgery is recommended for advanced lower rectal cancer patients, because the timing of cell death induced by radiation is delayed [58]. However, as mentioned above, the prolonged effects of combination of 1-MT and radiation were not evaluated.

## Conclusions

This is the first study that investigated the efficacy and underlying mechanisms of the combination of 1-MT and radiation both in vitro and in vivo. The nice thing of the current study is that radiation-induced activation of the Wnt/β-catenin pathway was effectively canceled by 1-MT in the combination treatment, as we expected. Apart from the modulation of the Wnt/β-catenin pathway, the combination of 1-MT and radiation and resulted in marked tumor suppression via multimodal mechanisms, including induction of cell cycle arrest, and enhancement of the antitumor immune responses. Here, we demonstrated an important example of targeting not only tumor cells but also tumor microenvironment in cancer treatment.

## Supplementary Information

**Additional file 1. **

## Data Availability

All data generated or analyzed during this study are included in this published article and its supplementary information files.

## Abbreviations

1-MT: 1-Methyltryptophan
7-AAD: 7-Aminoactinomycin D
AHR: Aryl hydrocarbon receptor
BrdU: Bromodeoxyuridine
BSA: Bovine serum albumin
CRT: Chemoradiation therapy
CT: Cycle threshold
CTLA-4: Cytotoxic T lymphocyte-associated antigen-4
DAPI: 4',6-Diamidino-2-phenylindole
FITC: Fluorescein isothiocyanate
Fr: Fraction
GAPDH: Glyceraldehyde 3-phosphate dehydrogenase
GCN2: General control non-depressive 2 kinase
HPF: High power field
IDO1: Indoleamine 2,3 dioxygenase 1
IFN: Interferon
Kyn: Kynurenine
KPM: Kynurenine pathway metabolites
LAT1: L-amino acid transporter 1
MTS: 3-(4,5-Dimethylthiazol-2-yl)-5-(3-carboxymethoxyphenyl)-2-(4-sulfophenyl)-2H-tetrazolium
MVD: Microvessel density
pCR: Pathological complete response
RT: Radiation therapy
RT-PCR: Reverse transcription polymerase chain reaction
SF: Survival fraction
siRNA: Small interfering RNA
Trp: Tryptophan
