# Supplementary material for: Enhancement of radiation therapy by indoleamine 2,3 dioxygenase 1 inhibition through multimodal mechanisms
Source: BMC Cancer. 2023 Jan 18;23:62. doi: 10.1186/s12885-023-10539-5 (PMC9847047; doi:10.1186/s12885-023-10539-5)
Supplement: Supplementary file 1 — Additional file 1. [file 12885_2023_10539_MOESM1_ESM.docx]

**S. Table 1**　Primer sequences.

| Species & Gene | Forward primer | Reverse primer |
| --- | --- | --- |
| Human *IDO1* | CACTTTGCTAAAGGCGCTGTTGGA | GGTTGCCTTTCCAGCCAGACAAAT |
| Mouse *IDO1* | CGGACTGAGAGGACACAGGTTAC | ACACATACGCCATGGTGATGTAC |
| Human *Axin2* | CAACACCAGGCGGAACGAA | GCCCAATAAGGAGTGTAAGGACT |
| Mouse *Axin2* | ATGAGTAGCGCCGTGTTAGTG | GGGCATAGGTTTGGTGGACT |
| Human *GAPDH* | AGGGCTGCTTTTAACTCTGGT | CCCCACTTGATTTTGGAGGGA |
| Mouse *GAPDH* | AATGGATTTGGACGCATTGGT | TTTGCACTGGTACGTGTTGAT |

*IDO1*: indoleamine 2,3 dioxygenase 1

*GAPDH* : glyceraldehyde 3-phosphate dehydrogenase


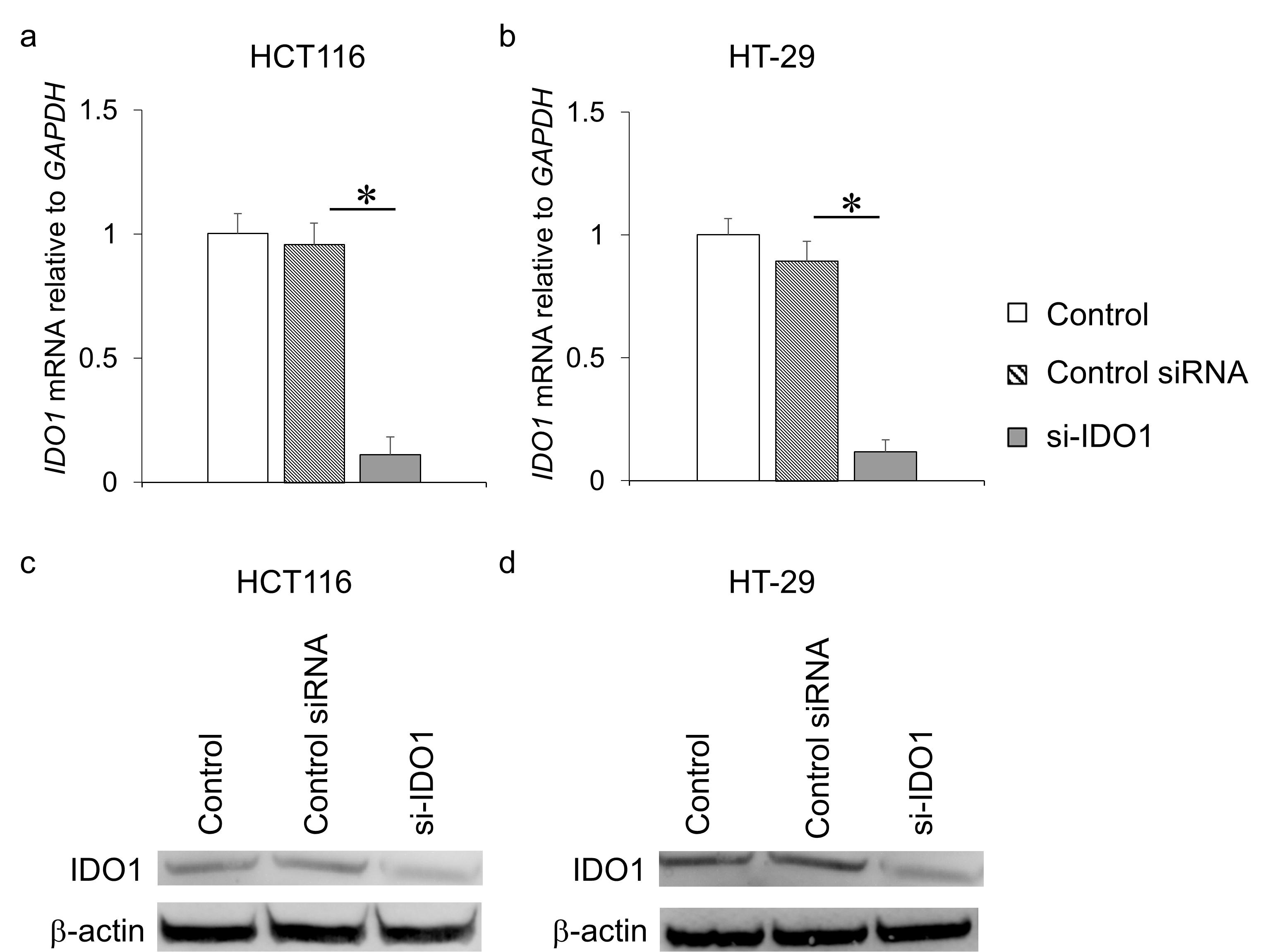


**S.Figure 1** Effects of IDO1-specific small interfering RNA (si-IDO1) on IDO1 mRNA and protein expressions. Relative expression of IDO1 mRNA in HCT116 (a) and HT-29 (b). Bars indicate standard deviations. * p < 0.001. IDO1 protein expression in HCT116 (c) and HT-29 (d).


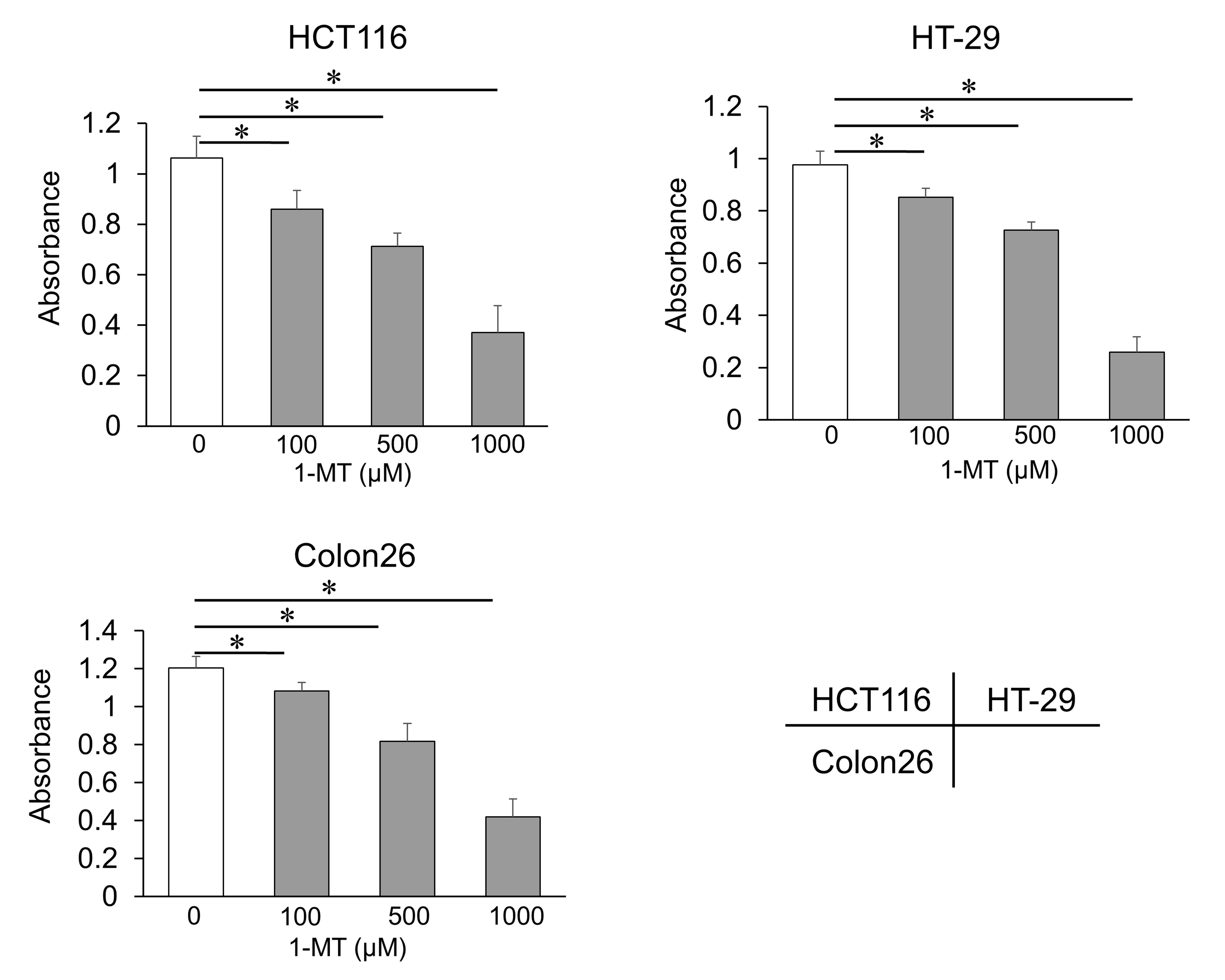


**S.Figure 2** Effects of IDO1 inhibition on the proliferation of colorectal cancer cells *in vitro.* Results after 96-h treatment are shown as absorbances measured at 490 nm by the MTS assay. HCT116 (upper left panel), HT-29 (upper right panel), and Colon26 (lower left panel). Bars indicate standard deviations. * p < 0.001.


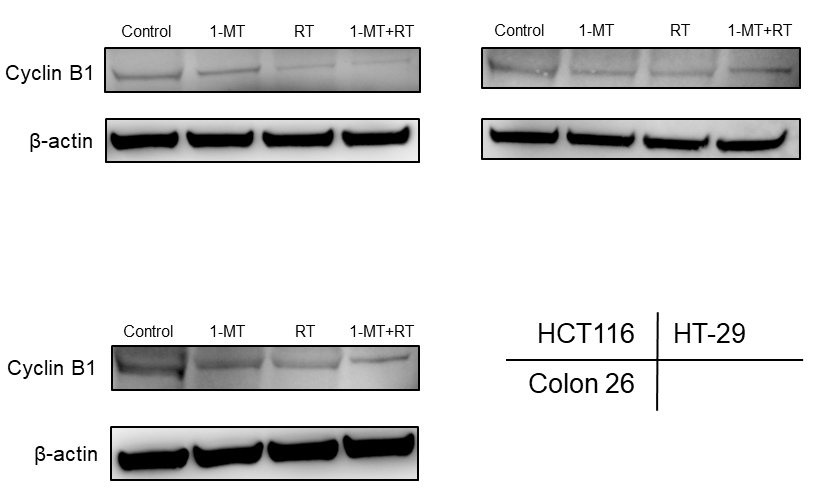


**S.Figure 3** Expression of cyclin B1 in colorectal cancer cells treated by 1-MT and/or radiation by western blot analysis. HCT116 (upper left panel), HT-29 (upper right panel), and Colon26 (lower left panel). RT: radiation. Results 48 h after administration of 1-MT (500 µM) and/or radiation (4 Gy) are shown.


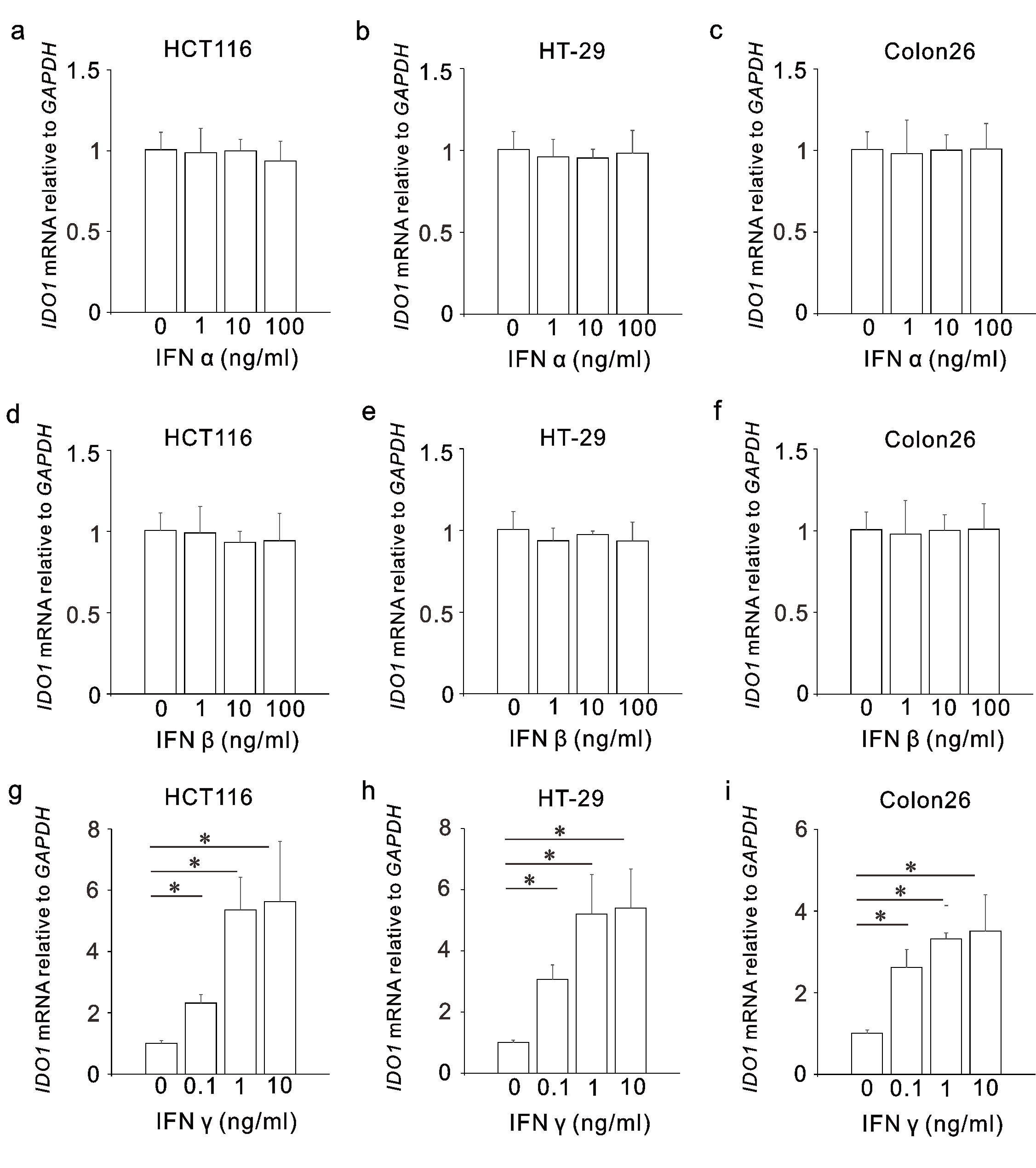


**S.Figure 4** Expression of IDO1 induced by IFNs in colorectal cancer cells. IDO1 expression treated by IFN α for 48 h is shown in (a) HCT116, (b) HT-29, and (c) Colon26. IDO1 expression treated by IFN β for 48 h is shown in (d) HCT116, (e) HT-29, and (f) Colon26. IDO1 expression treated by IFN γ for 48 h is shown in (g) HCT116, (h) HT-29, and (i) Colon26.


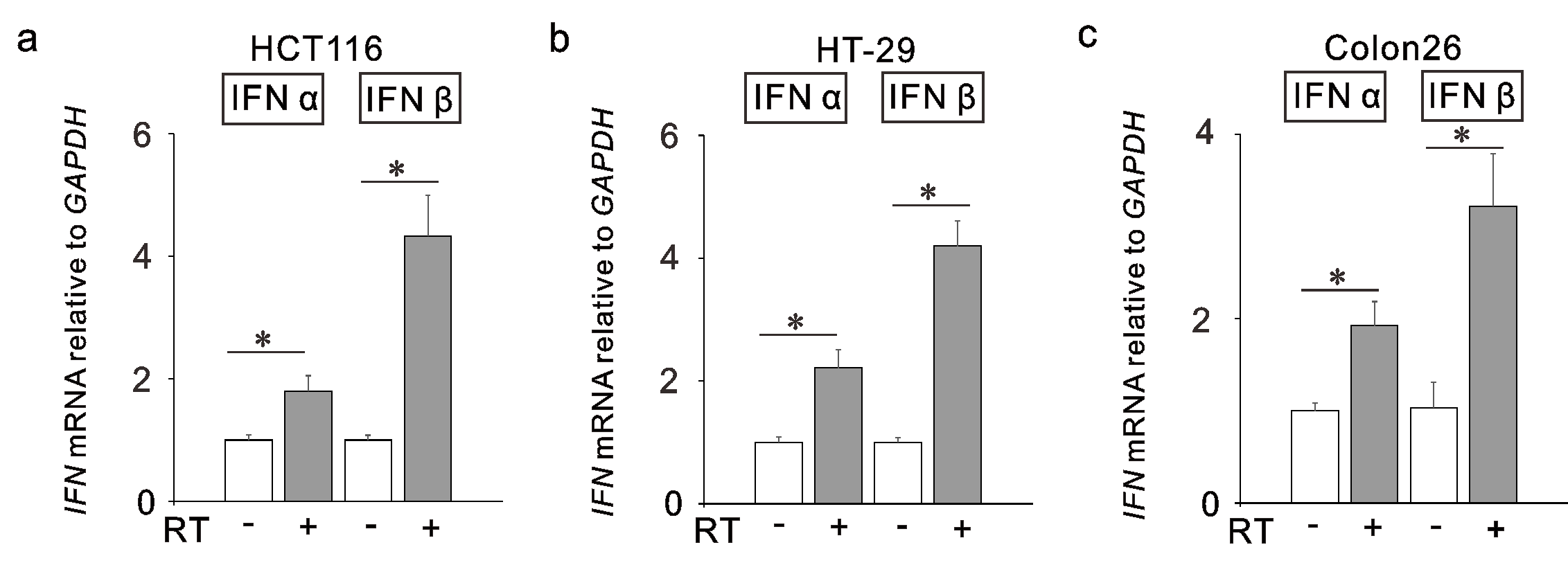


**S.Figure 5** Expressions of IFN α and IFN β after exposure to radiation. (a) HCT116, (b) HT-29, (c) Colon26. RT: radiation. Results 48 h after radiation (4 Gy) are shown.


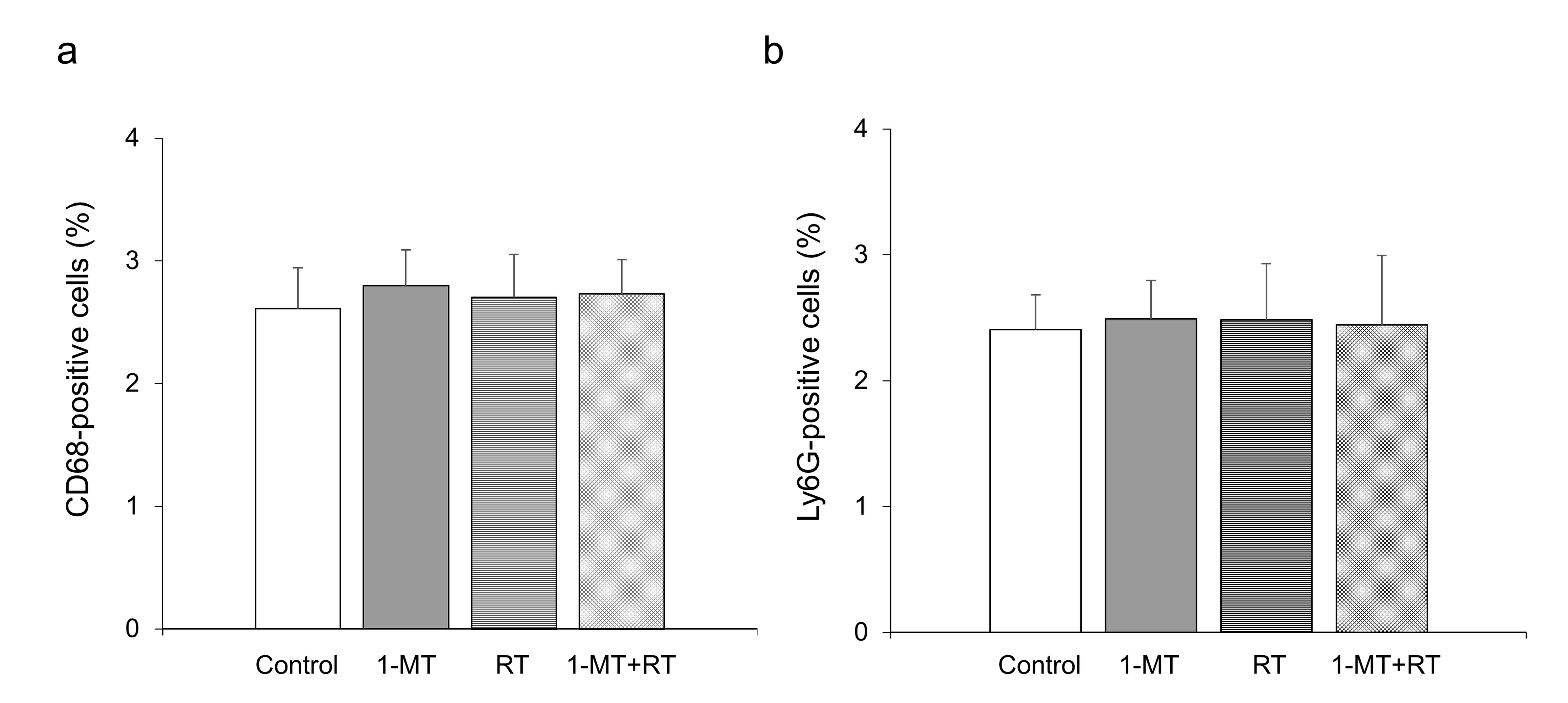


**S.Figure 6** Numbers of myeloid cells infiltrated into subcutaneous Colon26 tumor cells treated with 1-MT and/or radiation. (a) CD68+ macrophages (b) Ly6G+ granulocytes. RT: radiation.


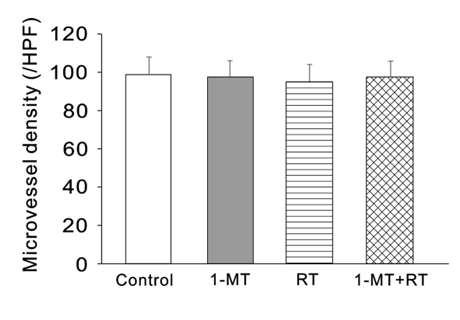


**S.Figure 7** Microvessel density of subcutaneous Colon26 tumors treated with 1-MT and/or radiation. RT: radiation. Bars indicate standard deviations.


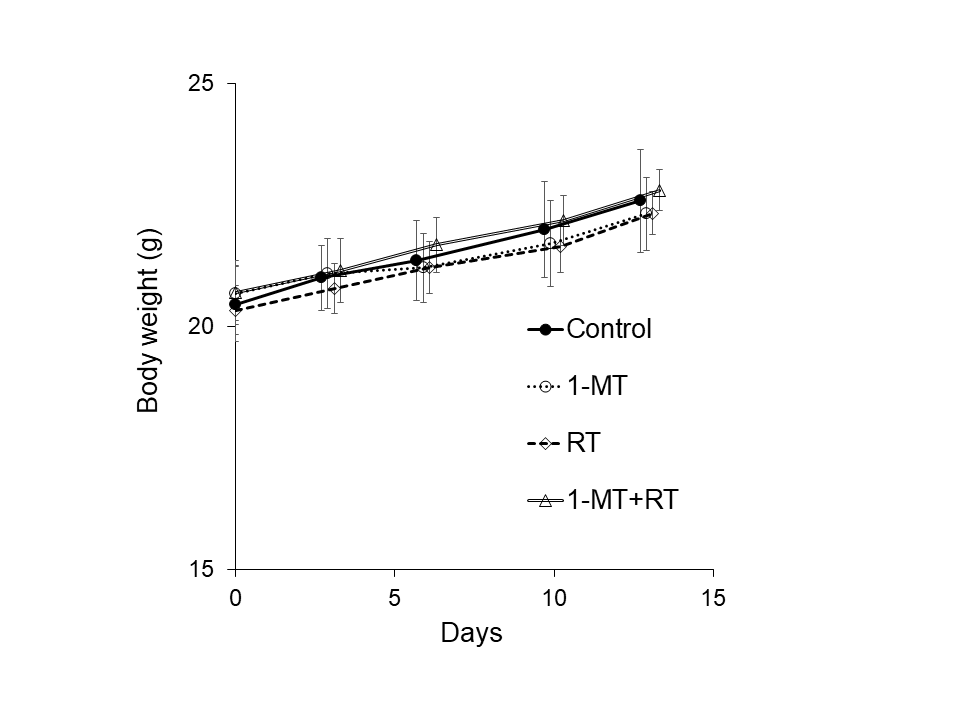


**S.Figure 8** Changes in body weight of Colon26 tumor-bearing mice treated with or without 1-MT and radiation. RT: radiation. Bars indicate standard deviations.


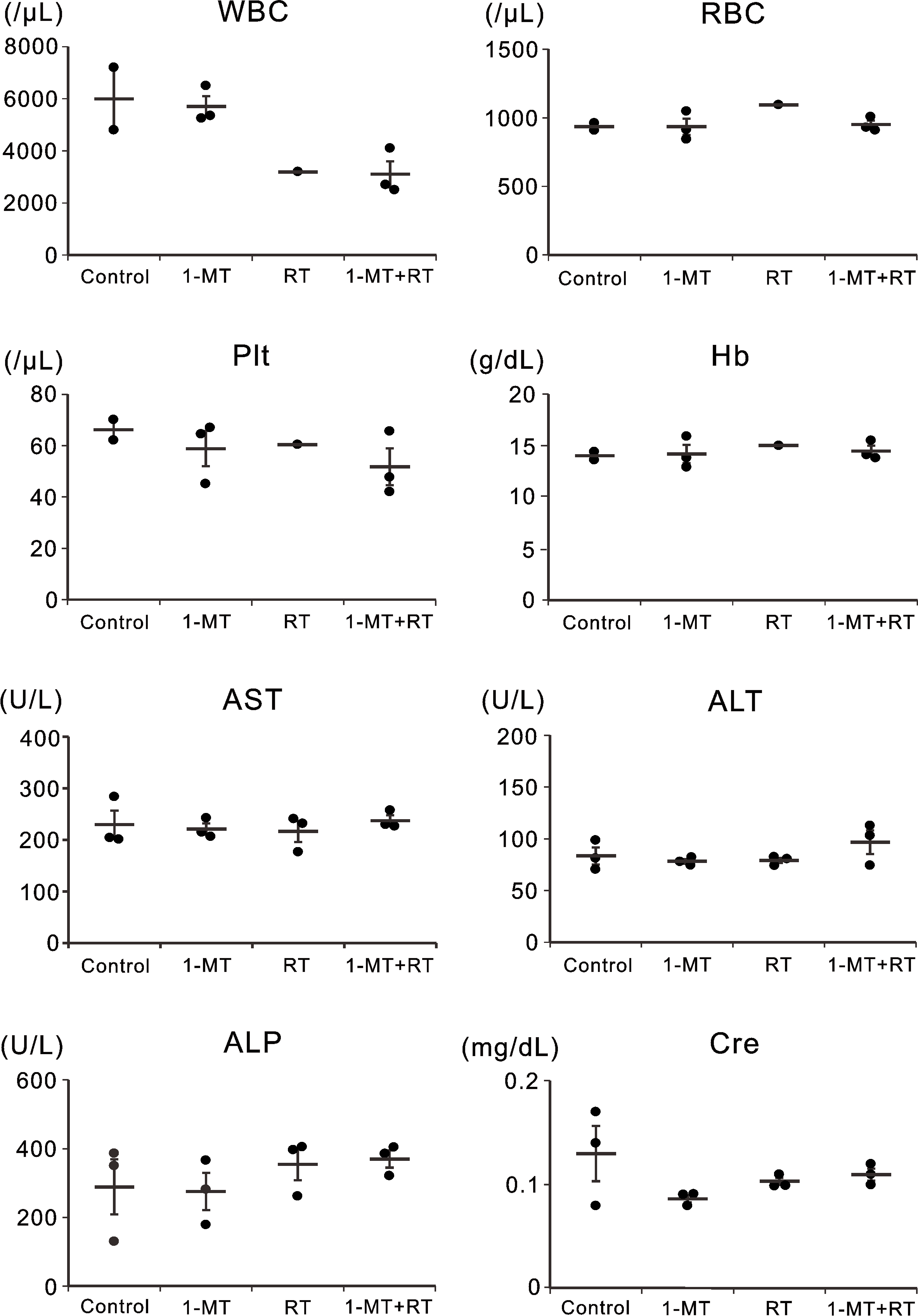


**S.Figure 9**  Blood test in mice treated with or without 1-MT and radiation. RT: radiation, WBC: white blood cells, RBC: red blood cells, Hb: hemoglobin, Plt: platelet, AST: aspartate aminotransferase, ALT: alanine aminotransferase, ALP: alkaline phosphatase, Cre: creatinine. Bars indicate standard deviations.


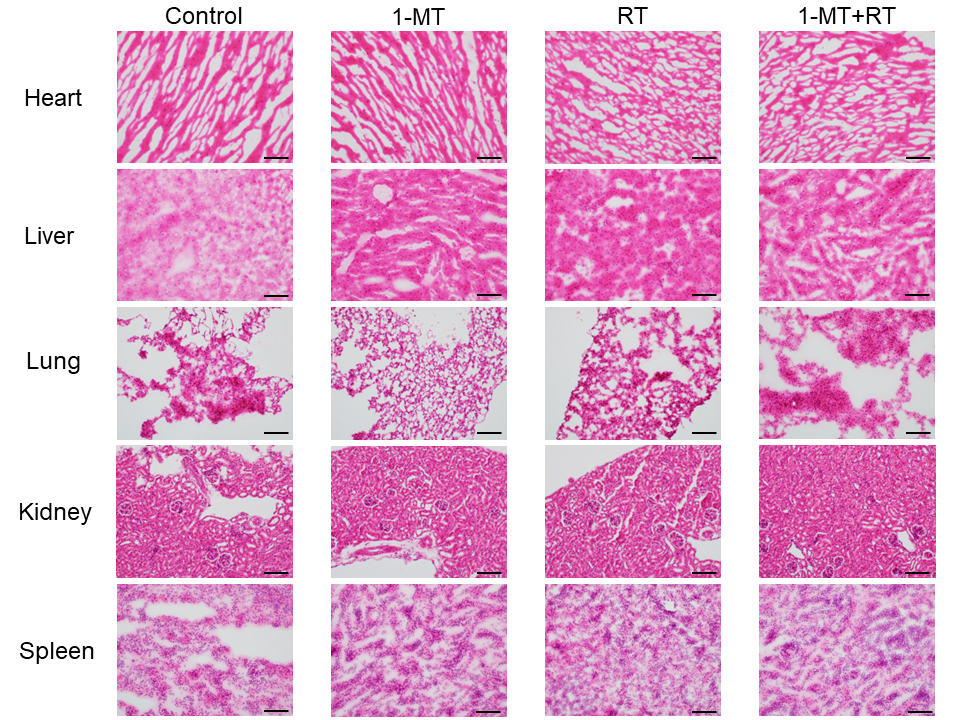


**S.Figure 10** Representative histological findings of organs in mice treated with or without 1-MT and radiation (hematoxylin-eosin staining. RT: radiation, Bars indicate 100 μm.


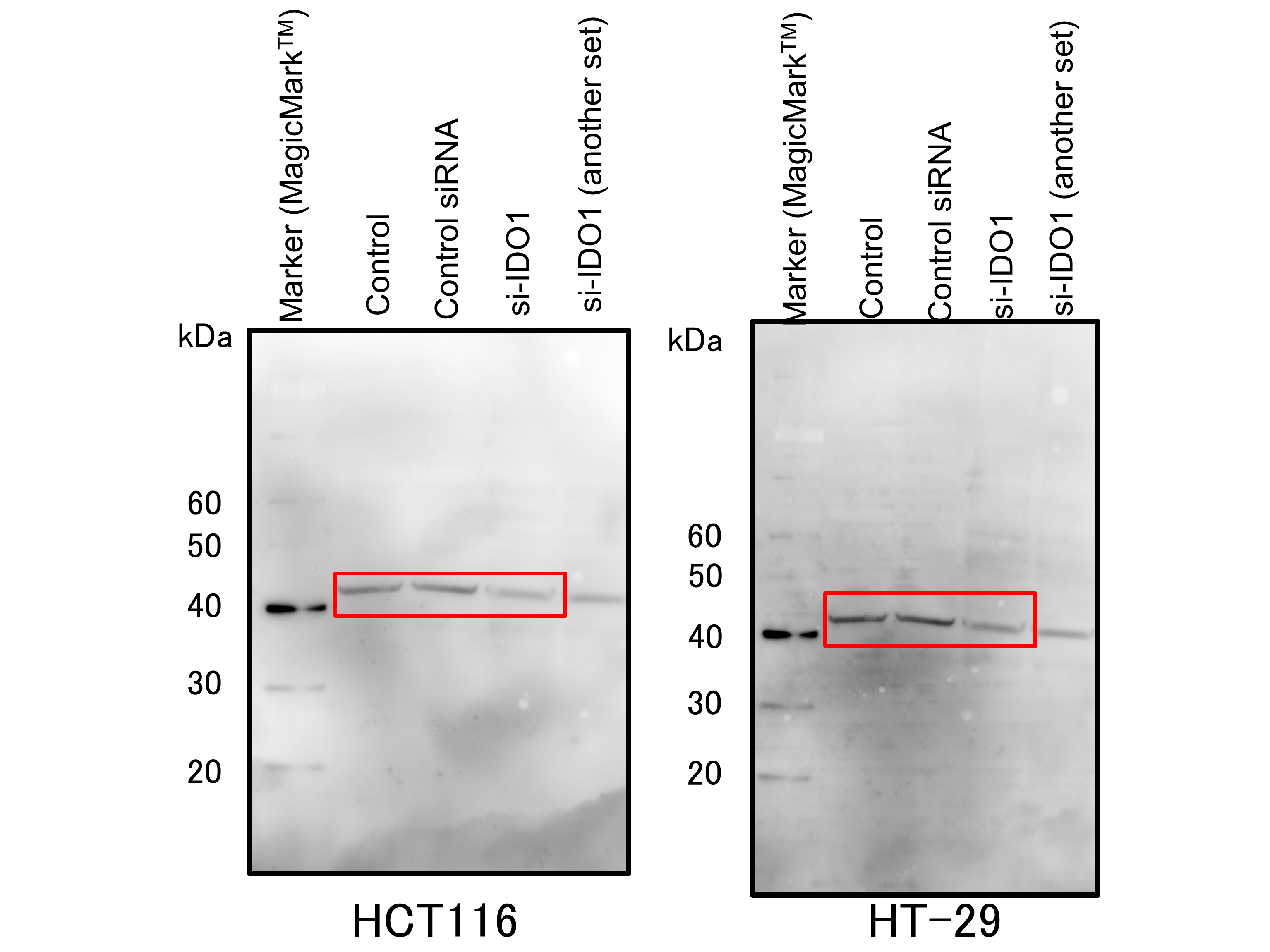

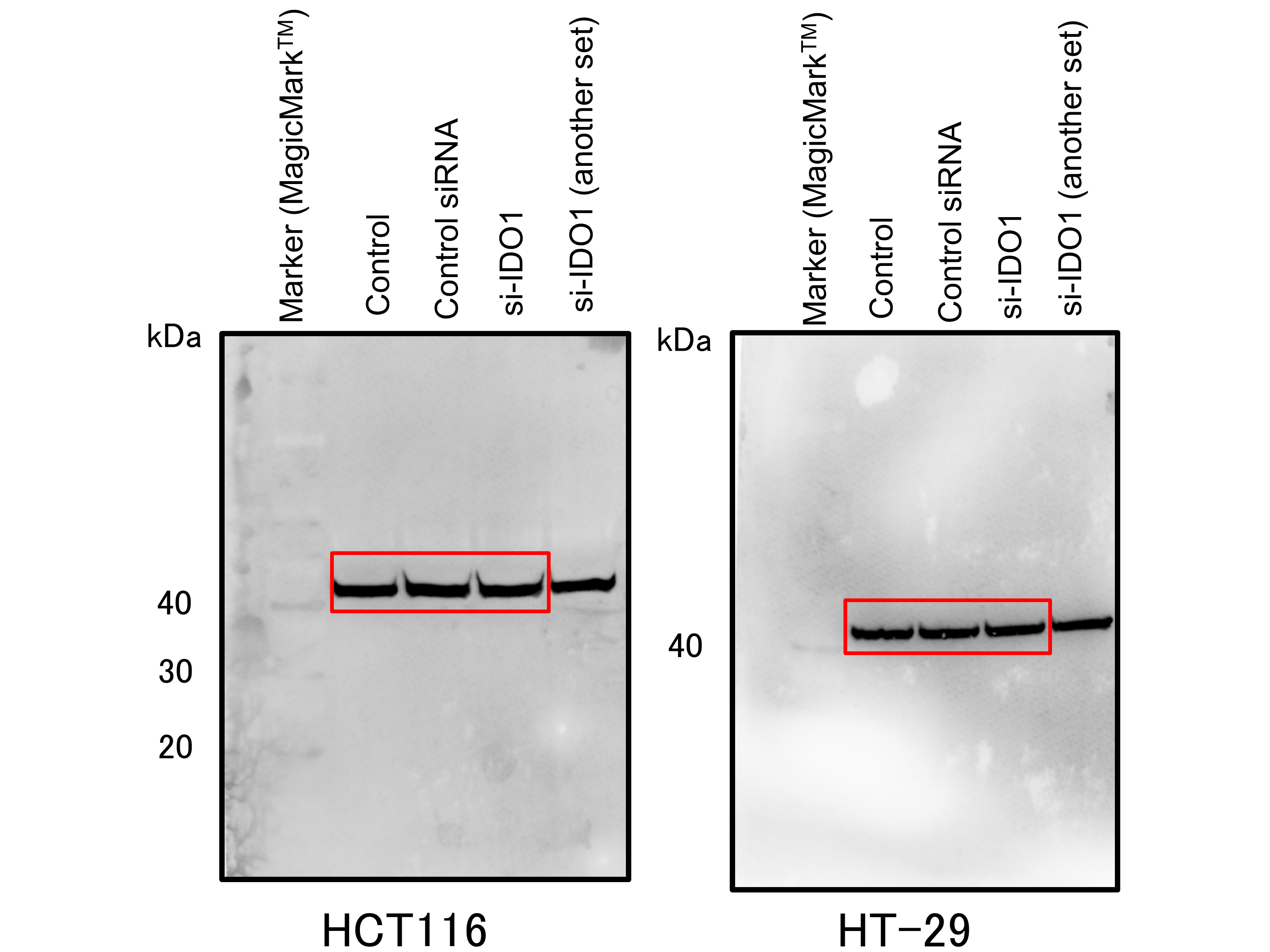


**Uncropped Images for S.Figure 1**


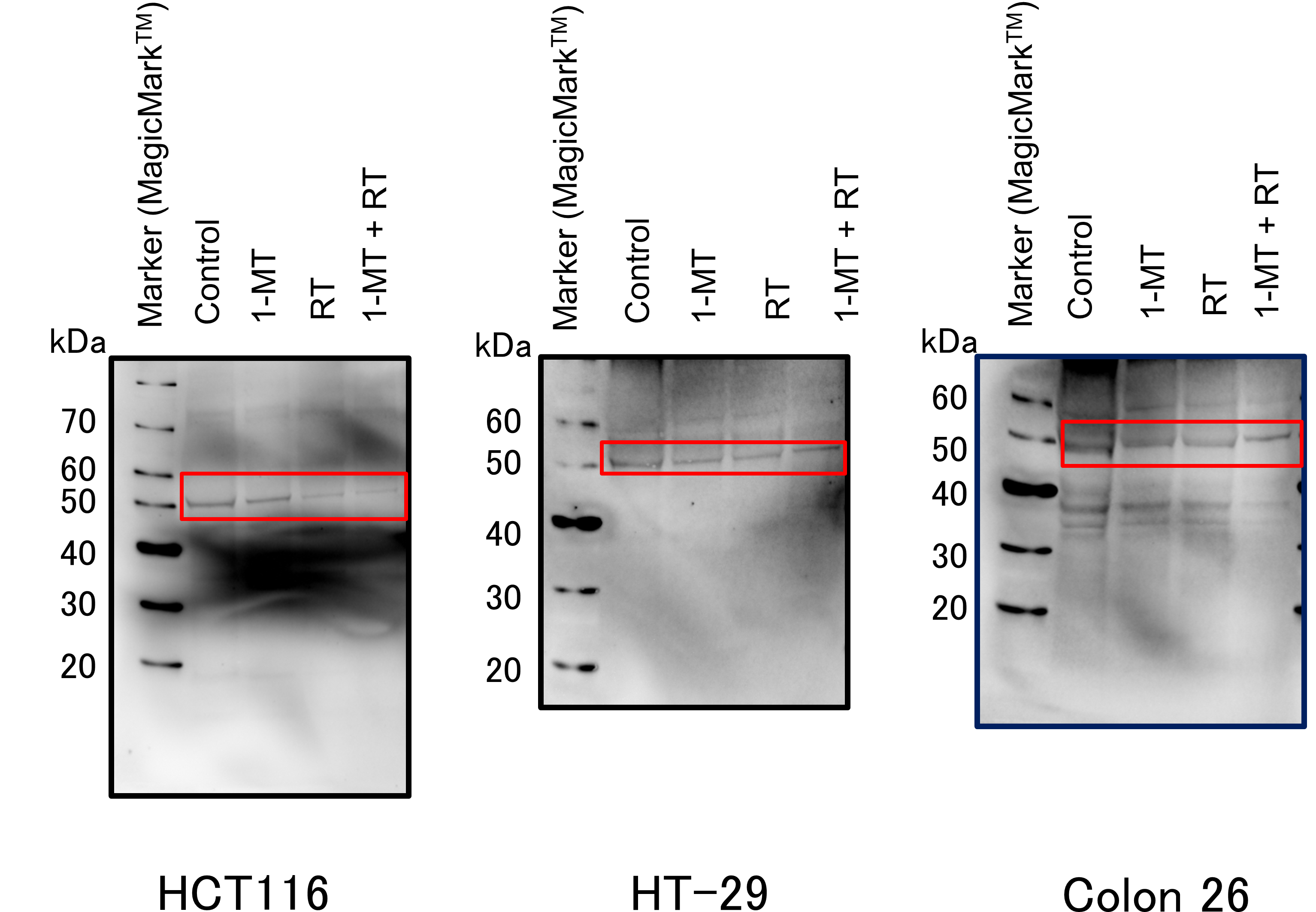

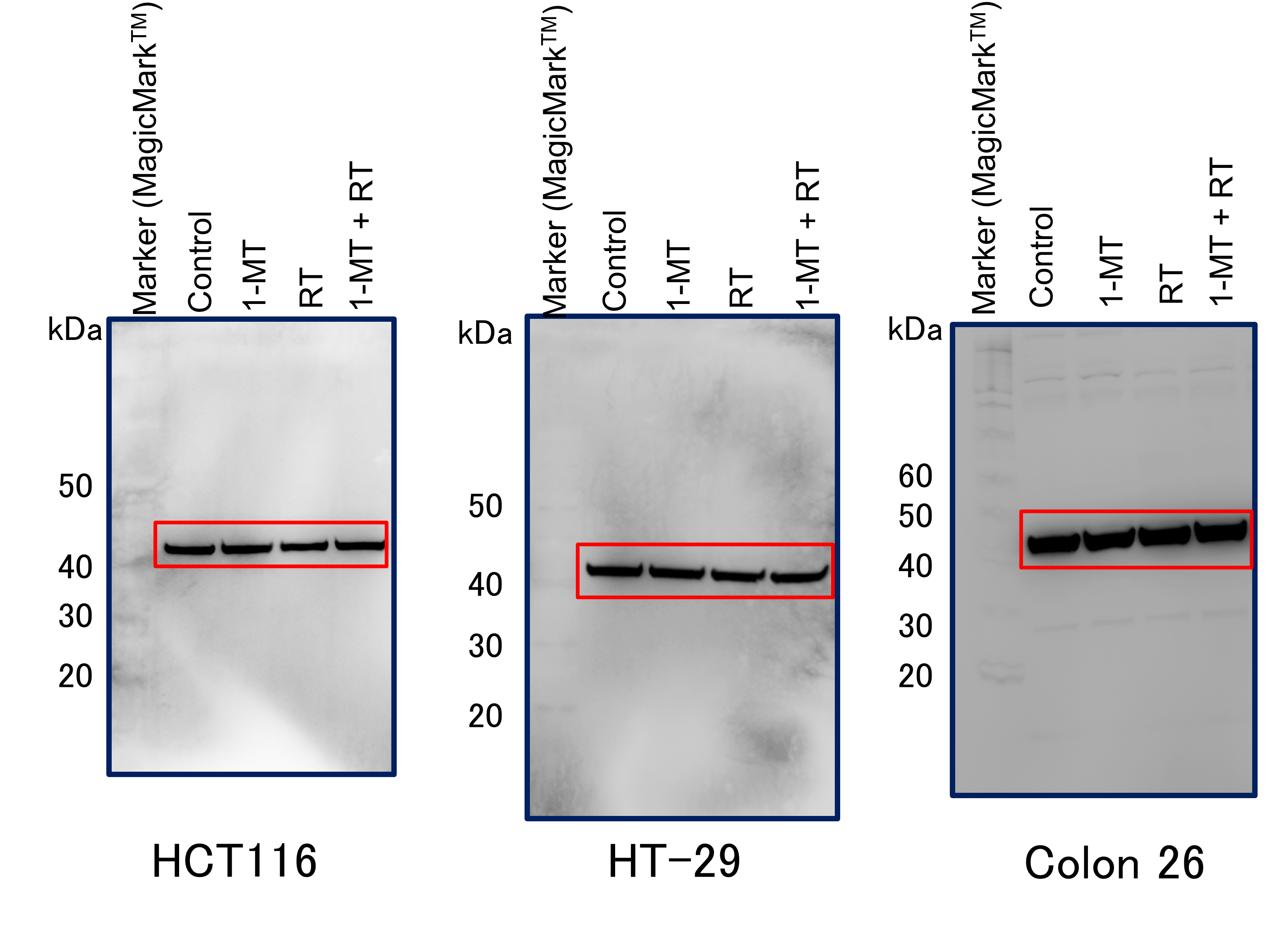


**Uncropped Images for S.Figure 3**
